# Skin-Inspired Ultra-Tough Supramolecular Multifunctional Hydrogel Electronic Skin for Human–Machine Interaction

**DOI:** 10.1007/s40820-023-01084-8

**Published:** 2023-04-13

**Authors:** Kun Chen, Kewei Liang, He Liu, Ruonan Liu, Yiying Liu, Sijia Zeng, Ye Tian

**Affiliations:** 1 College of Medicine and Biological Information Engineering, Northeastern University, Shenyang, 110169 People’s Republic of China; 2 Foshan Graduate School of Innovation, Northeastern University, Foshan, 528300 People’s Republic of China

**Keywords:** Ultra-tough hydrogel, Supramolecular, Flexible electronics, Knuckle training, Human–machine interaction

## Abstract

Multifunctional supramolecular ultra-tough bionic e-skin with unique durability for human–machine interaction in complex scenarios still remains challenging. Herein, we develop a skin-inspired ultra-tough e-skin with tunable mechanical properties by a physical cross-linking salting-freezing-thawing method. The gelling agent (*β*-Glycerophosphate sodium: Gp) induces the aggregation and binding of PVA molecular chains and thereby toughens them (stress up to 5.79 MPa, toughness up to 13.96 MJ m^−3^). Notably, due to molecular self-assembly, hydrogels can be fully recycled and reprocessed by direct heating (100 °C for a few seconds), and the tensile strength can still be maintained at about 100% after six recoveries. The hydrogel integrates transparency (> 60%), super toughness (up to 13.96 MJ m^−3^, bearing 1500 times of its own tensile weight), good antibacterial properties (*E. coli* and *S. aureus*), UV protection (Filtration: 80%–90%), high electrical conductivity (4.72 S m^−1^), anti-swelling and recyclability. The hydrogel can not only monitor daily physiological activities, but also be used for complex activities underwater and message encryption/decryption. We also used it to create a complete finger joint rehabilitation system with an interactive interface that dynamically presents the user’s health status. Our multifunctional electronic skin will have a profound impact on the future of new rehabilitation medical, human–machine interaction, VR/AR and the metaverse fields.

**Supplementary Information:**

The online version contains supplementary material available at 10.1007/s40820-023-01084-8.

## Highlights

- The skin-inspired multifunctional ultra-tough electronic skin with tunable mechanical properties was developed by a physically cross-linking salting-freezing-thawing method.
- The hydrogel integrates transparency (>60%), super toughness (up to 13.96 MJ m^−3^), good antibacterial properties (*E. coli* and *S. aureus*), UV protection (Filtration: 80%–90%), high electrical conductivity (4.72 S m^−1^), anti-swelling and recyclability.
- As a human–machine interface, it can be used for complex underwater activities, information encryption/decryption and finger joint rehabilitation training.

**Supplementary Information:**

The online version contains supplementary material available at 10.1007/s40820-023-01084-8.

## Graphical abstract

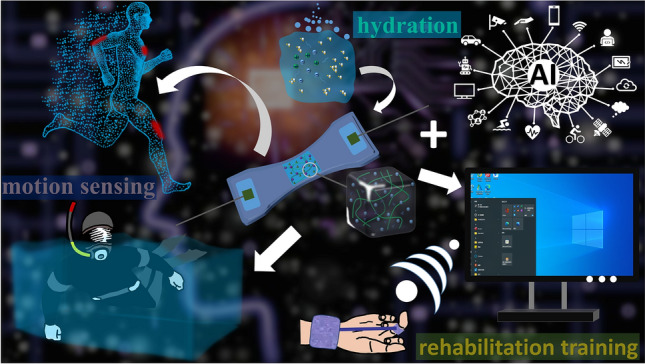

## Introduction

With the emerging of metaverse, human–machine interaction (HMI) attracts more and more attentions as the indispensable element in metaverse, even robotics, VR/AR and human–machine systems [1–4]. Current research on bio-organic interfaces has contributed significantly to the realization of HMI [5, 6]. As an important alternative, hydrogel electronic skin, as an emerging and promising human–machine interface, is endowed with sensibility [7, 8], flexibility [9], stretchability [10], comfortability [11, 12], biocompatibility [13] and scalability [14]. Therefore, hydrogel electronic skin enables people to seamlessly connect with electronic devices to achieve unique human–machine interaction and shows broad application prospects in future health monitoring [15], personal electronic equipment [16], intelligent robots [17, 18], wearable electronics [8, 19], tissue engineering [20, 21] and rehabilitation medicine [22, 23].

However, traditional hydrogel electronic skin can hardly load high-strength tasks due to the poor crosslink strength and mechanical strength of hydrogels, resulting in limited applications [24, 25]. To improve the mechanical strength, double networks [26], interpenetrating polymer network [27], heterogeneous structures [28] and radical copolymerization [29, 30] are introduced into hydrogels. But various chemical initiators or severe conditions (acid etching, ultrasonication, chemical initiation) [31–33] are required to make the hydrogel synthesis complex and non-green. Moreover, most bionic electronic skins, while enhancing the toughness by virtue of covalent bonding and effects, are without recyclability, which greatly limits the reusability of hydrogel e-skins as interfaces. In addition, the supramolecular structures can also be highly beneficial for the improvement of hydrogel properties. In general, 3D supramolecular gels constructed using amino acids [34], peptide groups [35] and self-synthesized [36] gelators can be adapted to a wide range of biological stimuli (pH, enzymes, etc.) [37, 38]. However, these gels are only moderately strong, soft and brittle, limiting their range of applications [39–41]. Recently, PVA-based supramolecular tough hydrogels are attractive, but most of them require the addition of rigid materials such as cellulose, clay nanosheets and graphene oxide [42, 43], or organic solvents (glycerol) [44] and involve more tedious chemical synthesis steps [45]. More importantly, their toughness is more dependent on the number and duration of freeze–thawing cycles [43, 46–49]. At the same time, most PVA-based bionic electronic skins have only a single functional feature [26] that cannot be adapted to the current needs for complex scenarios such as human–machine interaction devices, intelligent rehabilitation medicine, and underwater encrypted communication [50–52]. Therefore, there is an urgent need to develop a multifunctional and durable supramolecular hydrogel electronic skin interface which is ultra-tough, recyclable and appropriate for a variety of complex scenarios, with a simple and green method.

Here, we developed a skin-inspired ultra-tough and recyclable supramolecular hydrogel electronic skin, PVA-Gp/TA-CaCl_2_ (named: PGC), using the only physical cross-linking salting-freezing–thawing method without toxic by-products and complex process. We incorporated the salting method into the supramolecular self-assembly system to regulate the aggregated entanglement of macromolecular chains on molecules to obtain hydrogel networks with tunable mechanical properties. The salting agent we used is the sodium *β*-glycerophosphate (Gp), which is unused and more effective than commonly used salting agents (e.g., NaCl). Gp has strong hydration capacity and can make PVA molecules self-aggregate and entangle. We then accelerated the ionization process of Gp molecules by temperature and the hydrogel was also imparted with properties such as bacterial inhibition and UV protection by adding tannic acid (TA). Finally, one-time freeze-thawing was used to further increase the interchain crystallinity and thus improve its mechanical strength. Moreover, the mechanical strength of the hydrogel after the addition of Gp is 37 times higher than that of the hydrogel with the same mass of glycerol, and the tensile stress can reach 3.35 MPa. In addition, the concentrations of both Gp and PVA have a modulating effect on the mechanical strength of PGC hydrogels and can finally present a tensile strength of up to 5.79 MPa. Moreover, the hydrogel can be reused via recasting in a 100 °C water bath, and the mechanical stress can still be maintained at 100% after recycling six times. PGC hydrogels are endowed with super toughness, electrical conductivity, antibacterial, UV protection, anti-swelling, recyclability and transparency. The PGC hydrogel electronic skin can combine the protective properties of skin and flexible sensing for multiple complex scenarios in life. As a new generation electronic skin interface, the PGC hydrogel electronic skin can be used for precisely monitoring daily physiological activities (ECG, micro-expressions, joint activity and coughing) to achieve human–machine interaction. And the PGC hydrogel electronic skin was also used for underwater human–machine interaction via using underwater activity and the Morse code to transmit information, and a deciphering program for encryption/decryption of underwater/land-based information. In addition, the PGC hydrogel electronic skin was designed to be a knuckle rehabilitation tool for muscle strength training for knuckle-injury patients with Windows-system analysis software for dynamic simulation of muscle strength changes and feedback of health status, enabling the human–machine interaction (front-end data collection–-back-end analysis and processing). The multifunctional bionic electronic skin we have developed will have a significant impact on the future of flexible sensing, rehabilitation medicine, human–machine interaction, robotics, VR/AR and the metaverse.

## Experimental Section

### Materials

Polyvinyl alcohol (PVA, polymerization degree 1799, 99% Anhui Wanwei Co., Anhui, China), Calcium chloride (CaCl_2_, Aladdin), *β*-Glycerol phosphate disodium salt (Gp, > 95%, Aladdin), Tannic acid (TA, AR, MACKLIN), Glycerol (Shandong Dexinkang Medical Technology Co.). The deionized water for the experiment was produced by the ultra-pure water machine (UPTA-UV-20, Shanghai Shenfen Analytical Instrument, China) in our laboratory. Strains of *E. coli* (ATCC 25922) and *S. aureus* (ATCC 6538) were obtained from Beijing Baocang Biotechnological Company.

### Preparation of the PGC Hydrogel

The PVA (10 wt%) solid was added to deionized water and heated in a water bath at 100 °C for 0.5 h to obtain a clear solution. Subsequently, *β*-Glycerol phosphate disodium salt (Gp) with the same mass as PVA was added, and the water bath was again performed to obtain a bulk polymer. Next, a certain amount of TA (1 wt%) solution was added and the whole solution continued to be heated in a water bath to form the hydrogel. Then the mixed solution was put into a mold with a thickness of 1 mm, freezed for 12 h. Finally, it was soaked in 10 wt% CaCl_2_ solution for 10 min and the excess solution was wiped off the surface with absorbent paper. If not specified, use this scale hydrogel for human motion monitoring, underwater encrypted communication, joint rehabilitation training and other applications. As control test, we also prepared the pure PVA hydrogel, PVA-Gly (PGL), PVA-Gp (PGP), PVA-Gp-TA (PGT) hydrogel by the same process.

### Characterization

The hydrogel samples were analyzed qualitatively and quantitatively by Fourier Transform Infrared Spectrometer (VERTEX70, the scanning range is 400–4000 cm^−1^, From Brooke instruments, Germany). PVA, TA, Gp and other samples were also tested by it. The cross-linked PVA, TA, Gp and other samples were freeze-dried in a freeze-vacuum dryer (FD-A12N-80, Produced by Guansen Biotechnology, China) for 24 h. The hydrogel samples of PVA, PVA-Gp, PVA-Gp/TA-CaCl_2_ were observed by scanning electron microscope (SEM, Sigma300, Zeiss, Germany). The crosslinked hydrogel samples were also freeze-dried for 24 h in the freeze dryer (FD-A12N-80, Guansen Biotechnology, China), and then observed after spraying the gold. The acceleration voltage and magnification of the two control charts were 2–2.5 KV and 20,000 times.

### Mechanical Property Test

Tensile tester (5944, INSTRON, USA) was used to test the mechanical property. The test was carried out at a constant tensile speed of 100 mm min^−1^. All experimental samples were made into 30 mm × 15 mm × 3 mm. Tensile test was conducted at 25 °C and 50% humidity. The toughness of the hydrogel was obtained by integrating the area below the stress–strain curve. Fatigue resistance tests were performed on a mechanical tensile platform (JXLSPT-DBU, ZOLIX INSTRUMENTS, China) in the laboratory. Firstly, the initial length of the sample was noted down and the corresponding stretch length was set on the stretching platform. Finally, the ends of the sample were connected to the electrochemical workstation (CHI760E, Beijing Huake Putian Technology, China) for the stretching cycle experiment.

### Load-bearing Test of Hydrogel Webs

PGC fibers (diameter: 2 mm) were made in advance in the PET hose. The hydrogel web was woven from 6 PGC fibers (about 8 cm long). We dropped an orange of weight ∼ 172 g, from a height of 1 m, onto the hydrogel web. The falling process was clearly recorded by the camera.

### Electrical Property

The resistance change of hydrogel was tested by the electrochemical workstation (CHI760E, Beijing Huake Putian Technology, China). The AC voltage of the test resistance is 0.1 V, and the AC frequency is 1000 Hz. Then, the conductivity calculation formula is used $\sigma{ = }\;{\text{L/}}\left( {{\text{R}} \times {\text{S}}} \right)$, where ${\text{L}}$ (mm) represents the length of the hydrogel to be tested, that is, the distance between the two electrodes. ${\text{R}}$ and ${\text{S}}$ represent the resistance and cross-sectional area of the tested hydrogel, respectively. According to the resistance change of the hydrogel in the testing process, the resistance change rate trend of the whole process was calculated. $\Delta R/R_{0} { = }({\text{R}} - {\text{R}}_{0} )/R_{0} \times 100\%$ ($R_{0}$ represents the initial resistance when there is no change operation in the test, and $R$ is the real-time resistance in the test process).

### UV Protection Properties

The transmittance of the samples was measured in the wavelength range of 260–400 nm using UV–visible (vis) spectrophotometer (Lambda 650S, PerkinElmer, UK). All experimental samples sizes were 30 mm × 15 mm × 1 mm. Different concentrations of TA (mass ratio of TA (1): TA (2): TA (3) = 1:2:3) were used in the measurements. The conversion between transmittance and absorbance was done by the formula $A = - \lg T$ ($A$ represents the absorbance, and $T$ represents the transmittance in the test).

### In Vitro Antibacterial Study

We investigated the antibacterial activity of PGC hydrogels against Gram-negative and Gram-positive bacteria by agar diffusion assays using E. coli (ATCC 25922) and S. aureus (ATCC 6538) as model bacteria. The disc method allows visualization of the antibacterial effect of hydrogels. The E. coli and S. aureus were first inoculated in LuriaBertani broth culture (LB) separately and incubated at 36 °C for 24 h. 100 µL bacteria suspension was spread on the surface of LB plates and then the sterile hydrogel with the diameter of 6/8.5 mm (sterilized by ultraviolet irradiation) was placed onto the surface of the tablet. After incubation for 24 h at 36 °C, the growth of bacteria around the hydrogel was measured. We compared four different hydrogels of the same size and thickness, the size of the inhibition circle can be visualized. In order, 0–3 correspond to PVA, PGP, PGC and PGC_6_, respectively. Immediately after, we performed a quantitative analysis of the diameter of the inhibition circle. To further quantitatively assess the antimicrobial activity of prepared hydrogel, 3 mL of S. aureus and E. coli suspensions were added to the centrifugal tube containing sterilized PVA, PGP, PGC_3_, PGC_6_) hydrogel (1 g), respectively. After incubation for 24 h at 36 °C, a certain amount of each supernatant was taken for absorbance test in UV–visible (vis) spectrophotometer (TU-1900, Beijing Purkinje General Instrument, China). Bacterial suspensions without hydrogels were used as controls.

### Swelling Test

The prepared hydrogels were immersed in deionized water and simulated seawater (1 L of water dissolved 26.726 g NaCl), and the weight was measured at regular intervals. The dissolution ratio (SR) can be calculated according to the following formula: $SR = (W - W_{0} )/W_{0} \times 100\%$, where $W_{0}$ represents the weight of the original hydrogel and $W$ represents the weight of the hydrogel after swelling.

### Land/underwater Motion Monitoring

The PGC hydrogel was fabricated to a suitable size and then copper wire electrodes were connected to each end of the hydrogel to be used as a hydrogel sensor. The hydrogel sensor was attached to the body part to be monitored (e.g., finger and wrist) and encapsulated with a membrane (Fig. S1), and then connected to the electrochemical workstation (CHI760E, Beijing Huake Putian Technology, China) with wires at both ends. The resistance of the PGC sensor changed with the activity of the monitored part and was recorded in real-time in the electrochemical workstation, thus completing the data collection. For underwater motion monitoring, the pre-made PGC hydrogel needs to be soaked in deionized water or simulated seawater for a period of time, and then installed as a sensor according to the above method, it should be noted that the connection between the electrode and the hydrogel needs to be sealed with a waterproof film to exclude external interference. The sensor was attached to the finger to make different motion signals, and finally the data were recognized by the deciphering system to obtain valid information.

### Finger Joint Training Test

Molds with different sizes (diameter: 3 mm and 3.5 mm) were used to create PGC sensors of different thicknesses, which were then mounted into finger joint trainers and data were collected. The collected data were imported into MST analysis software for data processing, and finally the health status of this measurement was obtained.

## Results and Discussion

### Fabrication of Multifunctional PGC Hydrogels

We found β-sodium glycerophosphate (Gp), as a water-soluble sodium salt, can cause PVA solution rapid aggregation and even gelation of PVA molecules in a few seconds (Movie S1). As shown in Fig. S2, by adding the same mass of NaCl or Gp (10%, 20%, 25%), we found that Gp could cause PVA to aggregate more obviously. As the solubility of NaCl is limited at room temperature, NaCl cannot further accomplish the task of accelerating PVA aggregation at high concentrations. With the increase of Gp concentration, the aggregation phenomenon became more pronounced (Fig. S3). According to the Hofmeister theory (HT) [53], the gelation reaction of polymer molecules can be explained. Studies on ion-specific effects have shown that such phenomena are due to the effect of different ions on hydrated water around hydrophilic functional groups on hydrophobic chains [54–56]. When the salt ions reach a certain concentration, they can produce a strong hydration capacity. With the help of its own polarity, the salt ion adsorbs a large number of water molecules, thus precipitating the polymer molecules. For Gp, we propose the following chemical equilibrium equation [57–59]:

1 $$(({\text{HOCH}}_{2} )_{2} {\text{CHOPO(ONa)}}_{2} ) \leftrightarrow (({\text{HOCH}}_{2} )_{2} {\text{CHOPO(O}}^{ - } {)}_{2} + {\text{Na}}^{ + }$$

2 $$(({\text{HOCH}}_{2} )_{2} {\text{CHOPO(O}}^{ - } )_{2} + {\text{nH}}_{2} {\text{O}} \leftrightarrow \left[ {(({\text{HOCH}}_{2} )_{2} {\text{CHOPOO}}_{2} ({\text{H}}_{2} {\text{O}})_{{\text{n}}} } \right]^{2 - }$$

3 $${\text{Na}}^{ + } + {\text{mH}}_{2} {\text{O}} \leftrightarrow \left[ {{\text{Na}}({\text{H}}_{2} {\text{O}})_{{\text{m}}} } \right]^{ + }$$

**Fig. 1:**
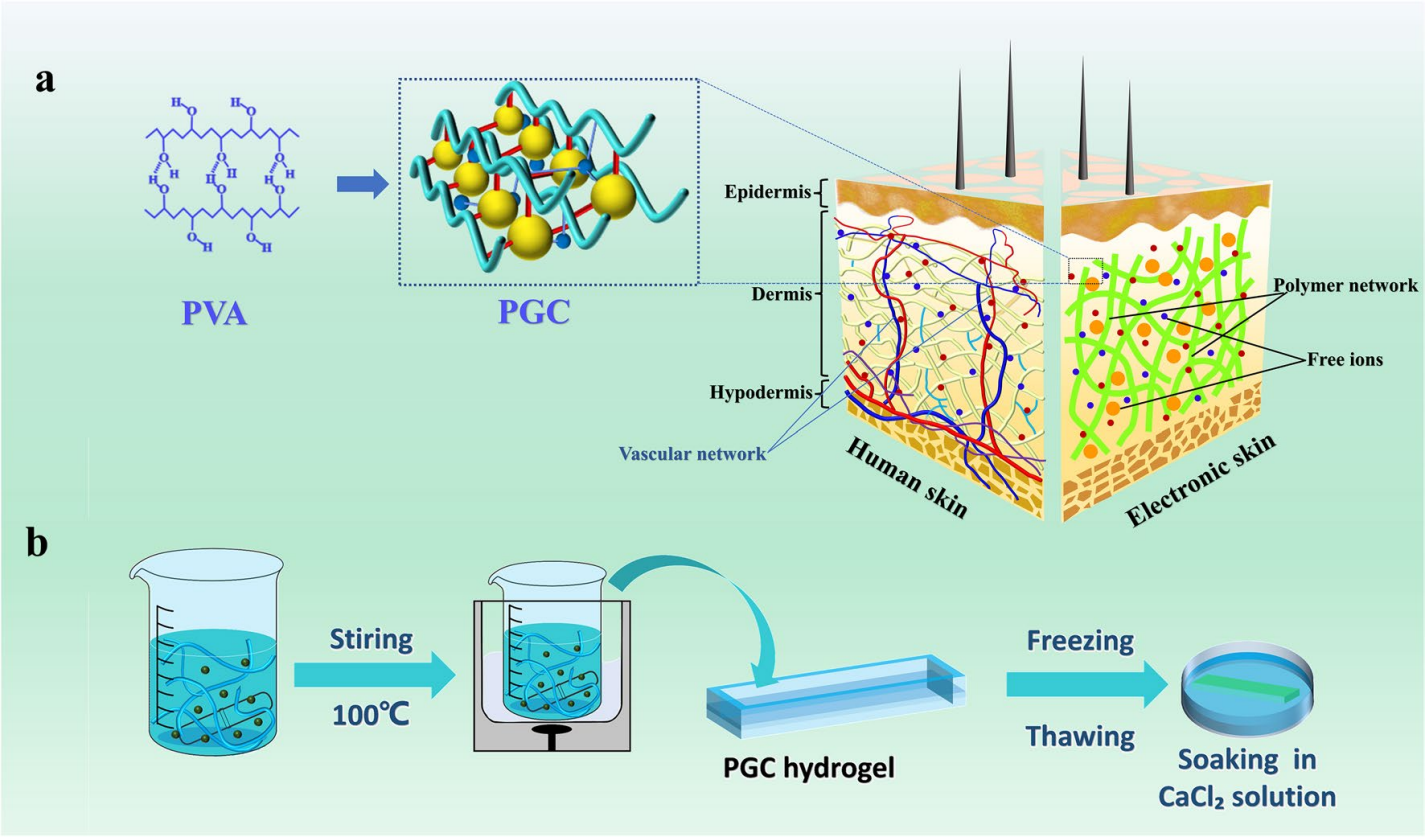
Structural properties of PGC supramolecular hydrogel. **a** Microscopic composition and skin-like properties of PGC hydrogel. **b** Production process of PGC hydrogel

Based on the above properties, we propose a new PVA-Gp/TA-CaCl_2_ (PGC) hydrogel patch using salting-freezing–thawing method (Fig. 1a). Inspired by the skin, we used PVA as the backbone to construct a tight bionic skin structure through the interaction between PVA, Gp, TA and CaCl_2_. In detail, under the strong hydration properties of glycerol phosphate molecules, the original electrostatic equilibrium between PVA molecules and water molecules was quickly broken in aqueous solutions. The water dipole has a strong affinity for both anions and cations, and the anions and cations use the electric field formed between them and the water molecules to form a polarization phenomenon that binds the water molecules around them (Fig. S4). Subsequently, we accelerated the ionization process of Gp in water using temperature to accelerate the gelation process of PVA. In this process, the hydrogen bonds between PVA and water molecules were broken and the inter/intra chain hydrogen bonds between PVA chains were increased substantially. Furthermore, we introduced functional groups such as phenol and ketone groups by adding Tannic acid (TA) to give it more functional properties. One TA molecule has 25 hydroxyl and 10 carbonyl groups and these conditions allow TA to form more hydrogen bonds with PVA for better bonding. Through the above reactions, the self-assembly of PVA, Gp and TA is completed in solution. Figure 1b displays the complete synthetic route for the PGC hydrogel. Gp and TA were added to the aqueous PVA solution and the water bath was heated to accelerate the reaction. Afterwards, the crystalline regions between the PVA chains were increased by freeze-thawing. The result showed a comparative graph of the gelation phenomenon after sequential addition of Gp, TA to the PVA solution (Fig. S5), indicating the positive effect of Gp, TA on the gelation process of molecular self-assembly. The addition of TA formed more H-bonds with PVA, and gave the hydrogel skin-like UV protection and antibacterial properties at the same time. The mechanical properties were then further enhanced by freeze-thawing at -20 °C for 12 h. Finally, it was immersed in CaCl_2_ solution to obtain higher conductivity and the resultant supramolecular hydrogel electronic skin was produced.

### Mechanical Properties of Supramolecular Hydrogel Electronic Skin

**Fig. 2:**
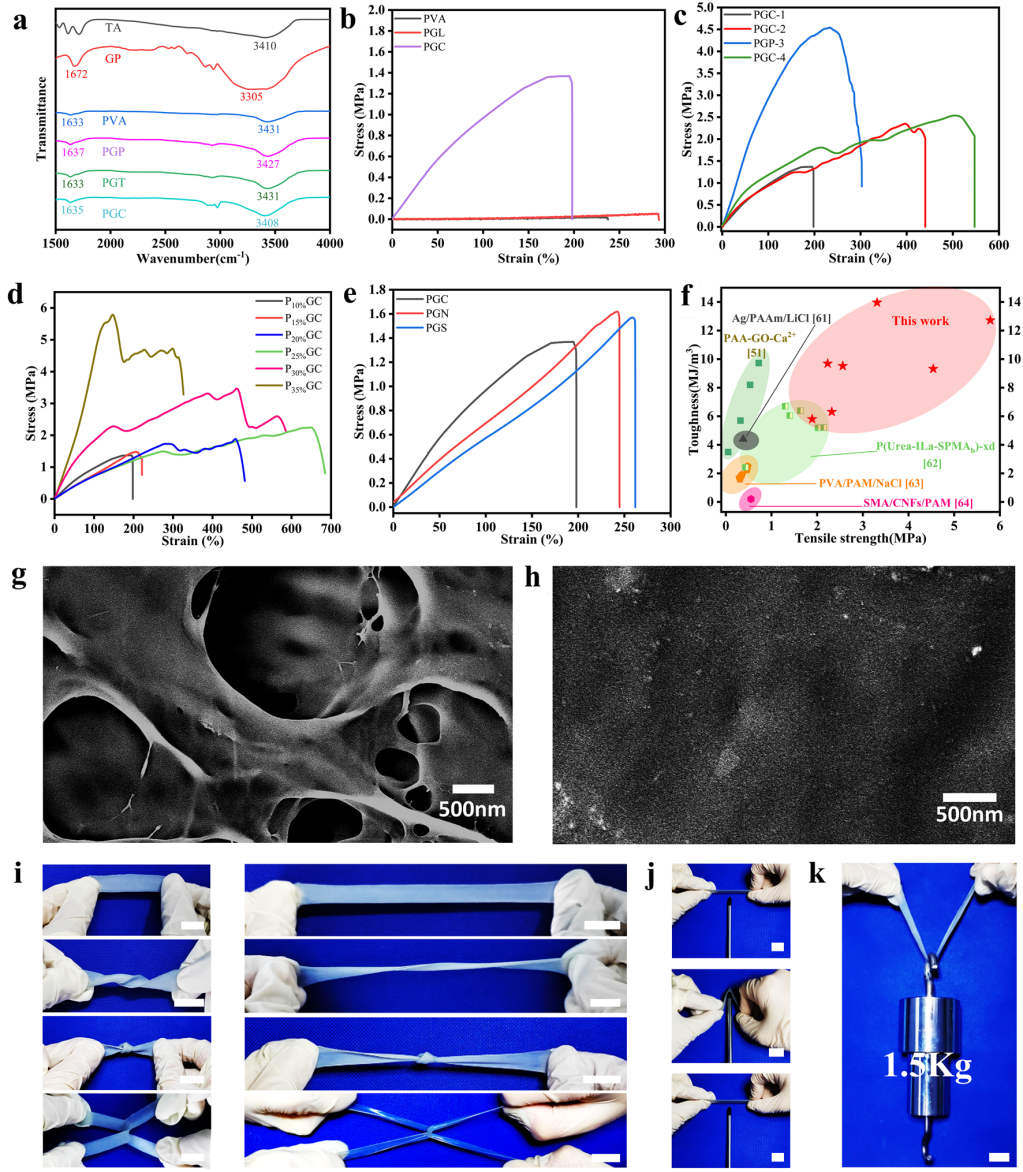
Characterization of PGC hydrogel. Different hydrogels of **a** FTIR spectra and **b** tensile curves. **c** Strain–stress curves of PGC hydrogel with different Gp concentrations. **d** Strain–stress curves of PGC hydrogel with different PVA concentrations (10%, 15%, 20%, 25%, 30%, 35%). **e** Immersion in different ionic solutions for the same time (CaCl_2_, NaCl, sodium citrate). **f** Comparison plots of the hydrogel in this work with other tough/supramolecular hydrogels by toughness versus tensile strength. SEM images of **g** PVA and **h** PGC hydrogels at same magnifications. Tensile deformation including: **i** stretching, twisting stretching, knotting stretching, crossing stretching and **j** puncture resistance. **k** Lifting an object 1500 times heavier than itself without damage. Scale bars: 2 cm in (**i–k**)

The supramolecular hydrogel electronic skin shows unique mechanical properties due to the strong molecular bonds. To better confirm the molecular bonds, FTIR investigation was conducted. As shown in Fig. 2a, the typical peaks of the TA appear at 3410 cm^−1^. The absorption peaks of Gp appear at 1672 and 3305 cm^−1^. For PVA, the peaks at 1633 and 3431 cm^−1^ can be assigned to the C = O tensile vibration of carbonyl and the O–H stretching vibration, respectively. The peaks at 3427 cm^−1^ of PVA-Gp (PGP) and 3431 cm^−1^ of PVA-Gp-TA (PGT) become sharper and more obvious after the addition of Gp and TA, which indicate that there is H-bond interaction among them. FTIR results can provide a theoretical basis for the interpretation of strong mechanical properties.

Figure 2b illustrates the tensile curves of different hydrogels. The tensile strength of pure PVA hydrogel is around 0.016 MPa, and by adding glycerol with the same mass fraction as PVA, the tensile strength of PVA-Gly (PGL) is increased to around 0.05 MPa. More importantly, when the glycerin is replaced with the same mass of Gp, the tensile strength of PGC reaches 1.36 MPa, which is nearly 27 times higher than that of PGL and nearly 85 times higher than that of pure PVA. It also exceeds the gel formed by the addition of a common salting agent (NaCl) by a factor of 5 (Fig. S6). These indicate that the toughening effect of Gp is more obvious than the usual salting agent (NaCl) or organic solvent (glycerol). Similarly, we next investigated the effect of different contents of Gp and PVA on the mechanical properties of the PGC hydrogel. Figure 2c illustrates the mechanical properties of the PGC hydrogels with different masses of Gp added. With the increase of Gp concentration, the tensile strength of PGC hydrogels showed a trend of increasing and then decreasing, and reached a maximum value of 4.54 MPa at PGC-3, which proves that Gp has a positive effect on the improvement of its mechanical properties. As shown in Fig. 2d, the tensile strength of PGC hydrogel gradually increased with increasing PVA concentration and reached a stress peak of 5.79 MPa at 35 wt%. In contrast, the 35% pure PVA only had a stress peak of 1.37 MPa, indicating that the tensile strength was not overly dependent on the high concentration of PVA (Fig. S7). Furthermore, we further calculated the toughness of the different gels mentioned above, and P_30%_GC showed an excellent toughness of 13.96 MJ m^−3^ (Fig. S8). The above results indicate that Gp and PVA play an important role in regulating the mechanical properties of PGC, and also make the PGC hydrogel have a wide mechanical tunable range.

In addition, we also investigated the changes of mechanical properties of PGC hydrogels soaked in different ionic solutions (Fig. 2e), and the best toughening effect was observed in comparison with NaCl solution, but due to balance the difference between mechanical properties and electrical conductivity, we chose PGC hydrogels soaked in CaCl_2_ as the object of further study. The study of the three ionic solutions also provides appropriate solutions for different needs in the future. In addition, we also verified the mechanical properties of PGC hydrogels after recycling, and the results demonstrated their tensile strength was maintained at about 100% after recycling six times (Fig. S9). The differences between the number of cycles may be due to the water uptake/loss of the gel system during the recasting process. Similarly, the compressive strain curve shows that PGC hydrogel also shows unique compressive properties and Gp can improve the compressive properties of hydrogels (Fig. S10). We further compared our work with other supramolecular/PVA-based gels and with the work on bionic e-skins in terms of mechanical strength and toughness (Fig. 2f and Table S1) [50, 60–63].

Further, we also verified the enhanced mechanical properties of PGC hydrogels at the microscopic level by SEM. Figure 2g shows the microstructure of PVA hydrogels after lyophilization, which has large pores. At the same magnification, the PGC hydrogel shows almost no pore structure, which indicates the improvement of the microstructure by Gp and TA (Fig. 2h). Meanwhile, the enhancement of its mechanical properties was further verified at the microscopic level. We also performed stretching, twisting stretching, knotting stretching and crossing stretching (Fig. 2i) to further verify its excellent mechanical properties. Figure 2j illustrates that the hydrogel was tough enough to resist puncture with a screwdriver. In addition, the hydrogel could also hold a load of up to 1,500 g (1,500 times weight of its own weight) without any damage (Fig. 2k). Moreover, we wove a web using six PGC hydrogels with 3 mm diameter. Remarkably, as we drop an orange of weight ∼ 172 g from a height of 1 m onto the web, the orange can keep intact on the web and the tough web was unbroken (Fig. S11). The various results above show that PGC hydrogels have excellent mechanical properties.

### Conductivity of Supramolecular Hydrogel Electronic Skin

**Fig. 3:**
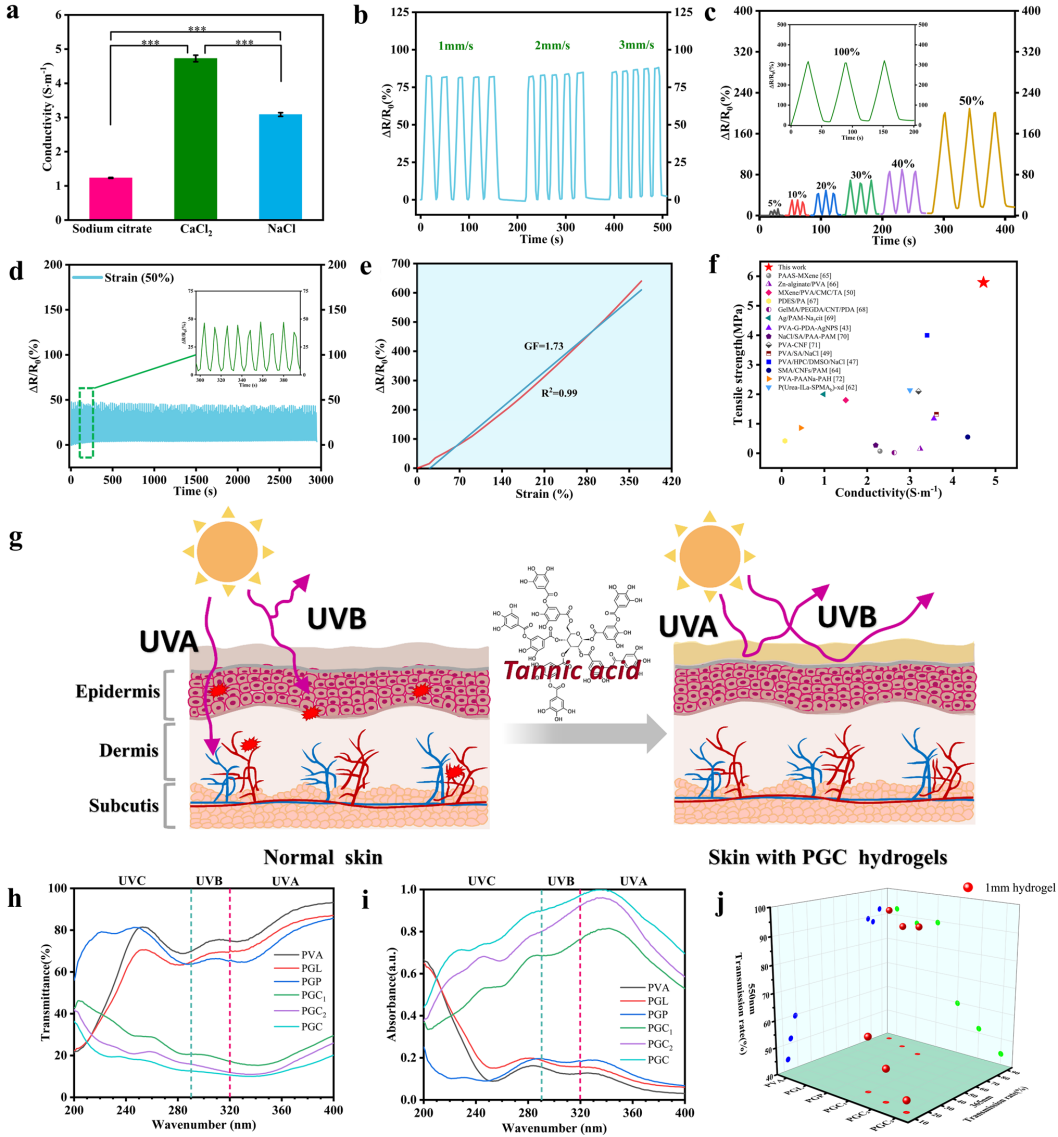
Conductivity and the UV shielding properties of PGC hydrogel. **a** Electrical conductivity of PGC hydrogels in different ionic solutions. **b** Cyclic stretching at different speeds. **c** Relative resistance changes under different cycling tensile strains at a fixed tensile speed of 2 mm s^−1^. **d** PGC hydrogel's antifatigue conductivity of tensile loading–unloading cycles in 3,000 s. **e** Gauge factor under the strain range of 0–370%. **f** Comparison plots of the hydrogel in this article with other recently reported works by tensile strength versus conductivity. **g** UV absorption diagram of PGC hydrogel. **h** UV–vis transmittance spectra, **i** UV–vis absorbance spectra, and **j** UV–vis transmittance at 550 and 365 nm of the PGC hydrogel with different TA contents. (***p ≤ 0.001)

The supramolecular hydrogel electronic skin also shows unique conductivity. First, we compared the conductivity of these different hydrogels to verify that Gp provides an abundance of free state ions to the hydrogel network (Fig. S12). The conductivity of the pure PVA hydrogel was maintained near 0.38 S m^−1^. After the addition of Gp, the conductivity was already > 1.1 S m^−1^, which was nearly three times higher than that of the PGL hydrogel, probably due to the presence of abundant free Na^+^. Similarly, it also shows that Gp has an elevating effect on conductivity in addition to regulating mechanical properties. We also tried to increase its conductivity with different salt solutions and finally found that the best results were obtained with CaCl_2_ (Fig. 3a). In addition, we also tested the conductivity of hydrogels immersed in three ionic solutions for different times (10, 20, and 30 min), respectively (Fig. S13). As shown in the figure, the electrical conductivity of the hydrogel increased with the increase of soaking time and reached 4.72 S m^−1^ after 10 min of soaking, which also laid the foundation for the excellent electrochemical sensing properties. Figure 3b shows the cycle test at different stretching speeds, the PGC can be stretched many times at different frequencies, which is greatly important for the precise detection. When the hydrogel sensor was exposed to various strains at a fixed strain speed of 2 mm s^−1^, the relative resistant changes gradually increased from 13% to 340% with the increasing strain from 5 to 100% (Fig. 3c).

To investigate the antifatigue conductivity, 270 cycling tensile loading–unloading tests were performed on the hydrogel sensor under 50% strain (Fig. 3d), and no obvious attenuation of electric signals was observed during the cycling process. The correlation between resistance change rates and stretch strains is depicted in Fig. 3e. The rate of change of resistance increased approximately linearly with the increase of tensile strain. The sensitivity (GF) was 1.73 with high linearity (*R*^2^ = 0.99), indicating that the strain is linearly related to the resistance change and can maintain a relatively stable state during the stretching process. We also compared GF with other hydrogel-based materials and showed high advantages (Table S2). We further compared the advantages of our hydrogel with other research work in terms of GF and electrical conductivity, and the results showed the superior performance of PGC hydrogels (Fig. 3f) [26, 43, 47, 49, 61, 63–71]. In addition, we tested the response/recovery time (100/150 ms) of the hydrogel sensor during stretching and compared it with other reported work (Fig. S14 and Table S3), showing a more prominent advantage. In a word, the high strain sensitivity and durability are essential for the long-term usage of hydrogel sensors.

### UV Protection of Supramolecular Hydrogel Electronic Skin

The supramolecular hydrogel electronic skin also demonstrates certain UV protection properties because the molecule of TA, a polyphenolic substance, contains abundant UV-absorbing functional groups such as phenol and keto groups and other chromophores (Fig. 3g). As shown in Fig. 3h, i, pure PVA, PGL and PGP had a weak absorption in the UV region, however, PGC showed a more pronounced absorption effect after the addition of a small amount of TA. And with the increase of TA content, the UV-blocking ability increased to a higher level, which can reach 90%. Figure 3j investigates the variation of transmittance at two specific wavelengths in the UV (365 nm) and non-UV regions (550 nm), respectively. Pure PVA hydrogel is transparent, but it has a light transmission of 87.9% at 365 nm, so it basically cannot filter UV radiation. More obviously, the UV transmittance decreased significantly when TA was added. And with the increase of TA content, the UV transmittance decreased from 77.9% at the beginning when no TA was added to 13%. This shows that PGC hydrogel has excellent UV absorption properties. Figure S15 shows more visually the variation in transparency of different hydrogel samples. The addition of TA changes the color of the hydrogel, but the writing behind the hydrogel can still be clearly seen. And with the increase of TA concentration, the PGC hydrogel can eventually maintain more than 70% light transmission.

### Antibacterial Properties of Supramolecular Hydrogel Electronic Skin

**Fig. 4:**
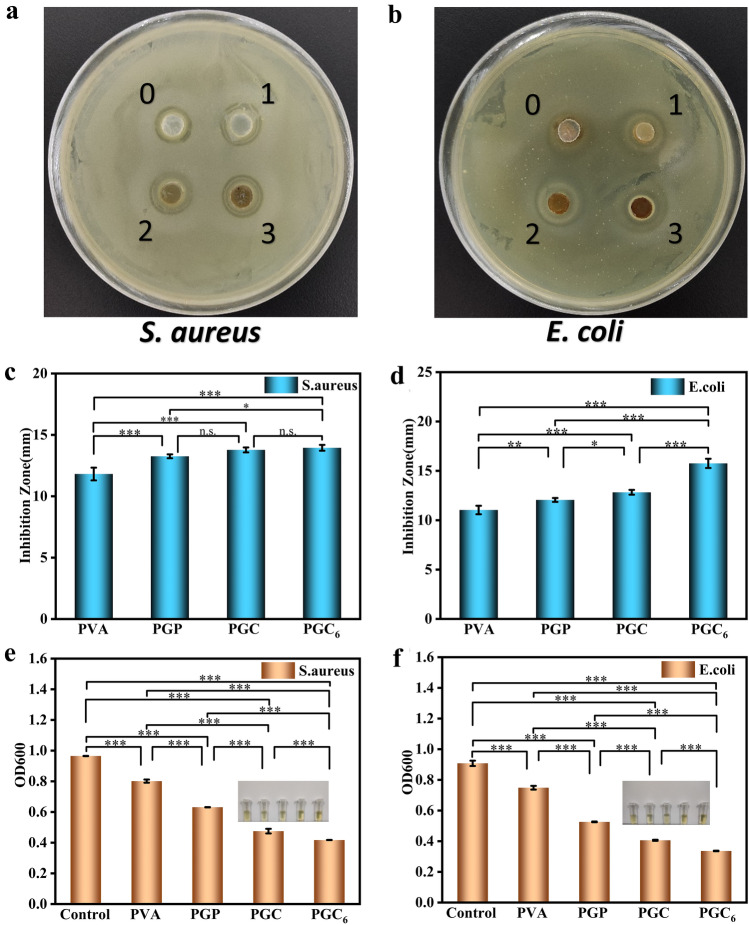
Antibacterial activity of PVA, PGP, PGC and PGC_6_ against **a** *S. aureus*, **b** *E. coli*. The diameter of inhibition circle of different hydrogels: **c** *S. aureus*, **d** *E. coli* (*n* = 5). The OD data of **e** *S. aureus*, **f** *E. coli*, the photographs in the figure are their corresponding bacterial suspension after 24 h incubation (*n* = 3). (*p ≤ 0.05, **p ≤ 0.01, ***p ≤ 0.001, n.s.: no significant difference)

The supramolecular hydrogel electronic skin also has unique antibacterial properties due to antibacterial and anti-inflammation properties of TA. The results showed that PVA and PGP hydrogel itself had some inhibitory effect on both *E. coli* and *S. aureus*. The addition of TA made the antibacterial effect more obvious (Fig. 4a, b), and the antibacterial effect kept increasing with the increase of TA content (Fig. 4c, d). Next, we conducted an absorbance test to reflect the antibacterial effect. We set up a bacterial stock solution blank control as well as several other control groups with concentration gradients and the optical density of the bacterial solution at 600 nm was measured (Fig. 4e, f). The results showed that the addition of TA could reduce the absorbance of the original bacterial solution. And the solution kept becoming clarified as the concentration of added TA increased, which was consistent with the results shown by the inhibition circle, indicating that the PGC hydrogel had a general inhibitory effect on both Gram-negative, positive bacteria. In addition, considering that the abundance of cations in PGP hydrogels may be partly responsible for the antibacterial activity, we also investigated the effect of their release behavior on the antibacterial repeatability (Fig. S16a, b), and the results showed that the inhibition effect slightly decreased with more repetitions, so it was confirmed that the release of cations would have an effect on the antibacterial repeatability. Therefore, it was confirmed that the release of cations has an effect on the reproducibility of antibacterial activity. Finally, we also verified the antibacterial repeatability and durability of PGC hydrogels (Figs. S16a, c and S17), and found that the PGC hydrogels had good antibacterial durability, however, the antibacterial effect decreased slightly with the increase of the number of repetitions, which might be related to the slow release of internal antibacterial substances.

### Human Motion Monitoring and Touch Screen Control

**Fig. 5:**
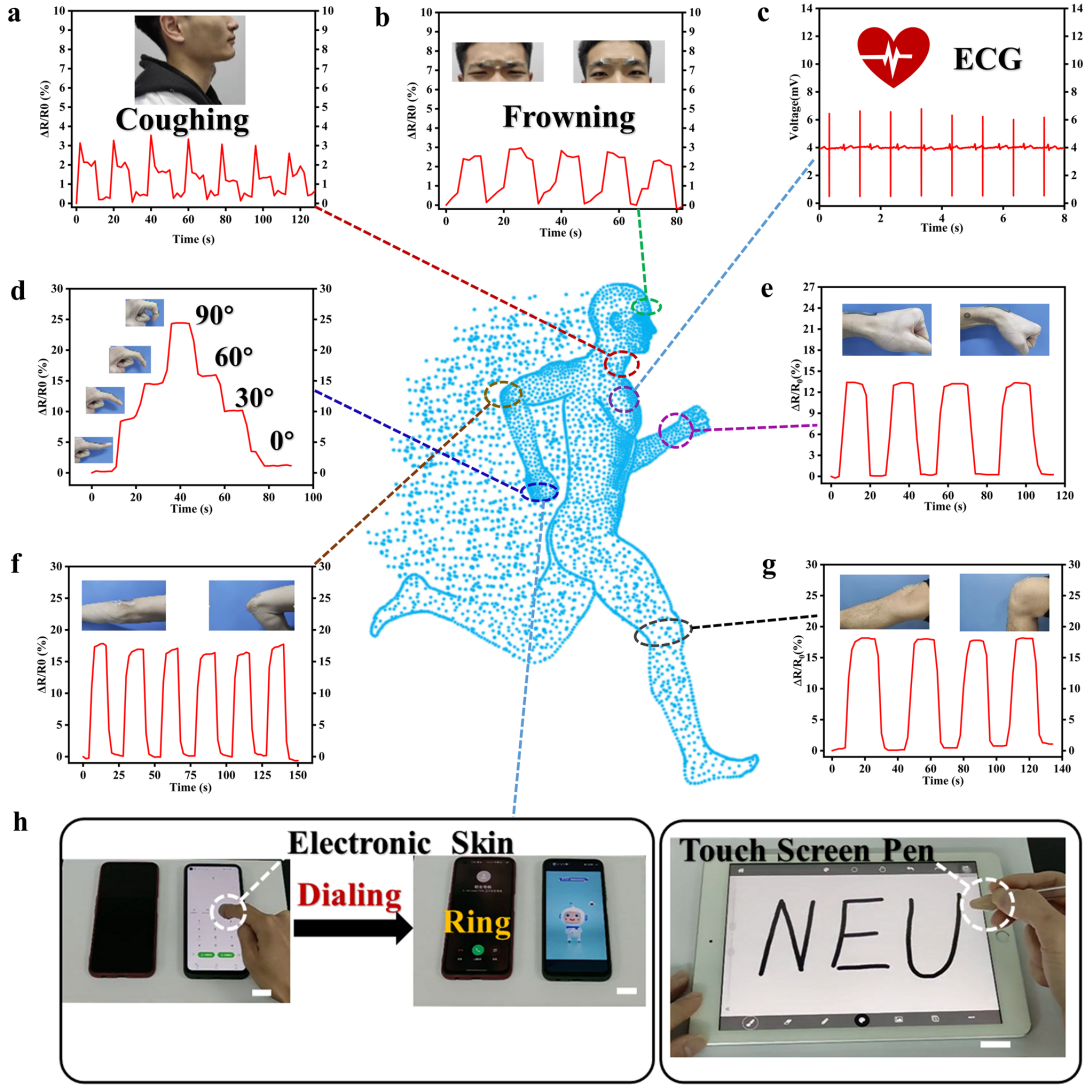
Responsiveness of PGC sensors to various life activities: **a** coughing; **b** frowning; **c** ECG monitoring; **d** finger bending with different angles (30°, 60° and 90°); The flexion movements of the **e** wrist, **f** elbow and **g** knee joints. **h** PGC hydrogel as electronic skin for human–machine interaction. Scale bars: 2 cm in **h**

The supramolecular hydrogel electronic skin with many properties such as toughness, conductivity, anti-bacteria and UV protection can be used for human movement monitoring and touch screen control. Due to the excellent electrochemical properties of PGC hydrogels, they can be used as flexible sensors to monitor various human movement behaviors. As shown in Fig. 5a, the PGC sensor was attached to the volunteer’s larynx and could detect changes in electrical signals when a cough occurred. Similarly, the sensor could monitor micro-expressions such as frowns (Fig. 5b). In addition, this sensor can be used as an electrode to obtain a stable heart rhythm output signal by using a heart rate monitoring device (Fig. 5c). In addition to responding to the above subtle signals, PGC sensors can also monitor larger strain signals. As depicted in Fig. 5d, the sensor steadily detected knuckle flexion and exhibited the ability to distinguish different bending extents (30°, 60° and 90°) of fingers.

Moreover, the sensors were sensitive to different joint movements such as wrist, elbow and knee (Fig. 5e–g). The flexion and extension of the joint corresponds to the change in amplitude of the response signal, and no significant changes in the limits were found in successive tests, verifying the stability and repeatability of the sensing in monitoring human activity. To further verify its human–machine interaction characteristics, we completed its information exchange with electronic devices: a finger/pen holder was made to control the touch screen, which can be easily operated for phone answering and writing (Fig. 5h). The electronic skin with the above-mentioned functional features can be extended to realize the construction of a new generation of human–machine interface for real-time health monitoring and wearable electronic devices.

### Underwater Information Encryption Transmission

**Fig. 6:**
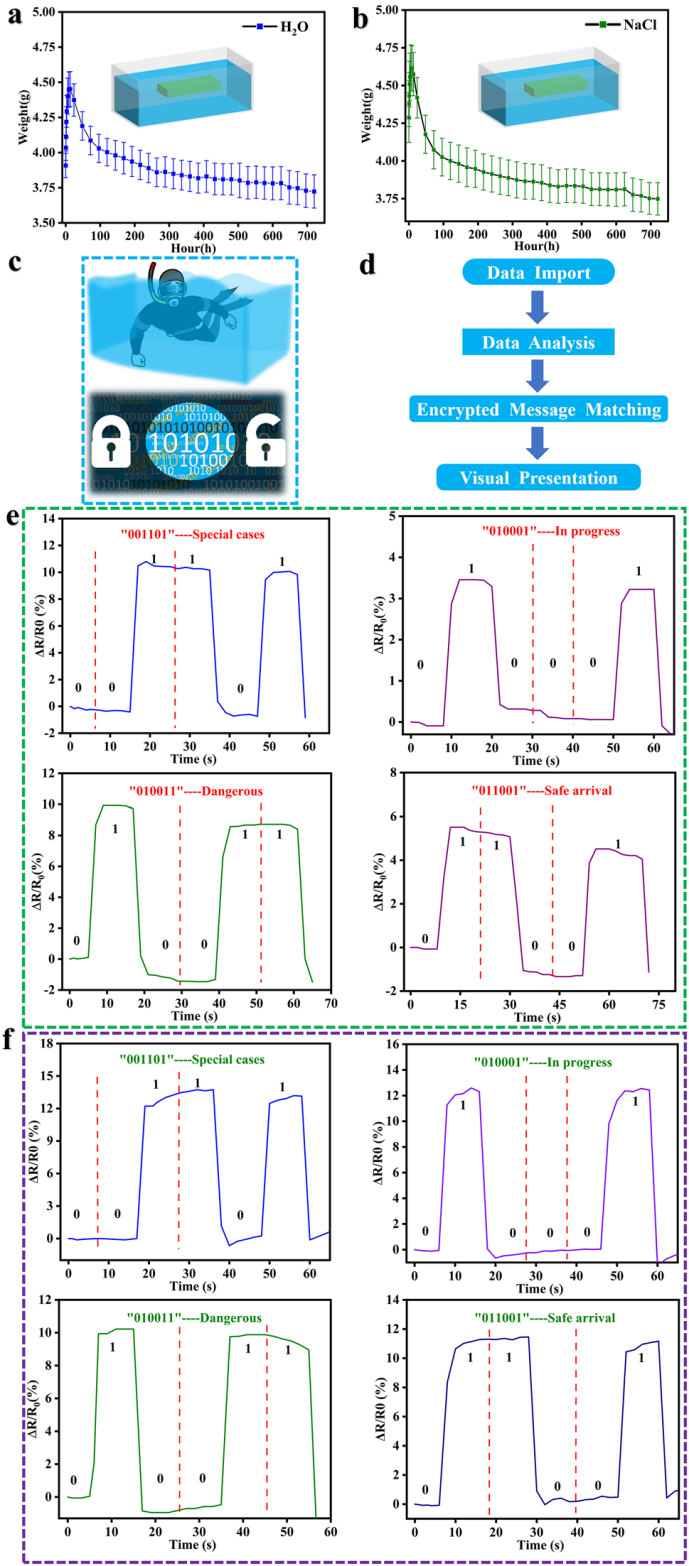
PGC hydrogel for underwater sensing applications. Swelling behavior of PGC hydrogels in **a** water and **b** simulated seawater. **c** PGC hydrogels for potential applications such as underwater or encrypted communications. **d** Operational framework of the code-breaking system. The operation of “001101”, “010011”, “010001” and “011001” in **e** water and **f** simulated seawater, respectively

The new generation of electronic skin must have bionic skin functional characteristics, so that it can be used in special environments such as underwater, wet, etc. As a result, we further verified the swelling characteristics of the PGC sensor in deionized water and simulated seawater to test its stability in underwater applications. According to the swelling of PGC hydrogels in deionized water and simulated seawater for 30 days, their shape and size remained essentially unchanged (Fig. S18). We determined the swelling characteristics of the hydrogel by measuring the change in weight. The swelling curve reflects the weight trend over 30 days (Fig. 6a, b), which shows that the weight of PGC hydrogel small increases and then decreases in deionized water and simulated seawater, and finally stabilizes. After 720 h, PGC could maintain more than 89% and 87% of its original weight in deionized water/simulated seawater, respectively. Therefore, PGC hydrogel sensors can be used for deep diving activities as well as for encrypted transmission of information.

When a diving athlete/swimmer wearing our sensors encounters an unexpected situation during underwater operations, he can use the Morse code to achieve onshore-underwater communication according to our pre-design (Fig. 6c). Then we also developed a code-breaking program for this purpose, and the flowchart shows the logical structure of the program (Fig. 6d). According to Fig. 6e, f, the PGC sensor renders four cryptographic communication states in water and simulated seawater, respectively. Taking advantage of the good anti-swelling properties of the PGC sensor, we wear it on the finger joint and make a series of bending movements according to the design of Morse code communication. Then, the front-end data acquisition device obtains stable waveform changes and collects real-time resistance change information, and then uses our code-breaking program to build a human–machine interface and draws real-time change data into images, and finally sends alerts to people (Movie S2).

In addition, the PGC sensor can be worn in other joint positions to determine the divers’ sports training status based on changes in the motion waveform. We designed 8 cryptographic states (Fig. S19), which can be added by users according to the actual situation. To realistically simulate various environmental issues in diving, we simulated humidity, waves, and other factors in an experimental environment and tested them in freshwater and simulated seawater (Fig. S20). The experiments show that the PGC still has excellent sensing characteristics under these conditions. In addition, we also tested the GF under wet and water flow conditions (Fig. S21), which is slightly improved compared to the GF under normal conditions, probably because the PGC absorbs water molecules and the internal conductive pathways are more active. In addition, the long-term stability of PGC strain sensors was demonstrated: no significant deterioration in sensing performance was felt even after 10 days of immersion in freshwater and simulated seawater, respectively (Fig. S22). As a result, PGC sensors can be used with wireless transmission devices to achieve land-submersible communication and human–machine information interaction. As a new generation of intelligent communication devices, it has significant application potential in civil/military deep diving.

### Finger Rehabilitation Training

Finger rehabilitation is an important part of rehabilitation medicine. In finger joint rehabilitation, the speed of recovery can be significantly enhanced by plyometric training. Therefore, we designed a rehabilitation training tool based on our supramolecular hydrogel electronic skin and developed a Windows-based easy-to-operate finger joint rehabilitation training analysis software (MST) to obtain a complete rehabilitation training system. This finger rehabilitation training system can analyze the collected data according to our algorithm model and finally present the health condition to the user in the most intuitive way to get a better user experience. During use, the user only needs to wear the sensor and perform the corresponding joint movement (Movie S3), and the subsequent operation process is easy.

**Fig. 7:**
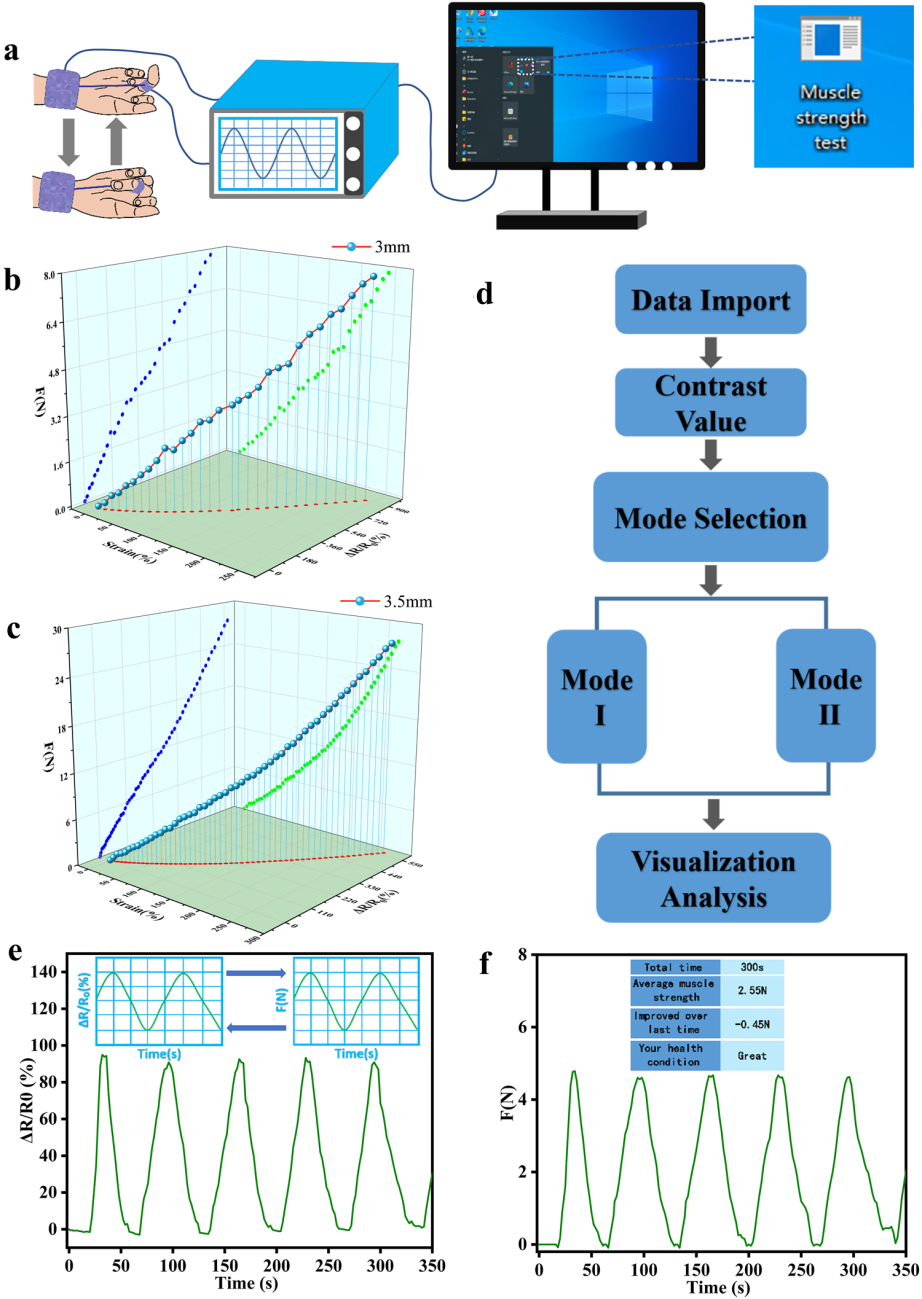
PGC sensor for finger joint muscle training. **a** Diagram of the training-analysis process. **b**, **c** Force-stress-resistance three-dimensional curve and resistance real-time change curve for 3/3.5 mm type sensor. **d** Flow chart of the operation of MST analysis software. Tester’s resistance rate of **e** change-time, **f** traction force–time curve

With the help of the built human–machine interaction platform, we can then monitor the entire recovery process in real time (Fig. 7a). We designed two sizes of PGC sensors (diameter: 3 and 3.5 mm) and constructed the corresponding curves of pulling force-strain-resistance change rate in a 3D chart (Fig. 7b, c). The projection of each data point on the three planes represented their values of pulling force, strain and resistance change, respectively. Subsequently, the obtained real-time data of the joint motion was transformed into a waveform plot of the rate of change of resistance/tension using the MST software. The waveform of tension reflects the change of muscle force. Finally, the software analyzes and compares the average muscle strength of the current measurement with the previous one, and communicates the information such as “total time” and “improved over last time” to the user visually (Fig. S23 and Movie S4).

In addition, patients can choose different sizes of sensors to improve muscle strength depending on their recovery status. Figure 7d illustrates the process framework of the software. Taking the 3.5 mm size sensor as an example, its original resistance change rate curve has the same trend as the tension change profile after data matching (Fig. 7e, f), so that the muscle force magnitude can be visualized. This system, developed in combination with the PGC sensor, achieves a close integration of rehabilitation medicine and human–machine interaction, and the system can also be combined with the Internet of Things system to achieve telemedicine. In addition, compared with previous work in terms of stress, conductivity, toughness, antimicrobial properties, UV resistance, recyclability, AI interactivity, and fabrication process (Tables S4, S5), our work shows that the overall more advantageous performance. Therefore, PGC sensors have important application potential in the future in the field of human–machine interaction-rehabilitation medicine, which is more focused on user experience.

## Conclusions

In conclusion, we have developed a skin-inspired and mechanically tunable ultra-tough hydrogel e-skin using a supramolecular system for a human–machine interaction interface. By adding a novel salting agent (Gp), molecular self-assembly is performed under the salting-freezing–thawing action, and finally a strong hydrogel with tunable mechanical properties is obtained. Gp enhanced the interchain interaction of PVA and we obtained a tough hydrogel with skin-like functional properties by adding TA. The hydrogel integrates ultra-toughness, transmission (> 60%), UV protection (Filtration: 80–90%), electrical conductivity (4.72 S m^−1^), antibacterial (*E. coli* and *S.* aureus), anti-swelling and strain sensitivity properties. And its physical cross-linking properties allow it to be recast multiple times and used multiple times. As an interactive interface, PGC can also be used in complex scenarios such as underwater sensing and message encrypted communication, in addition to monitoring physiological activity (ECG, micro-expressions and joint movements). We have also used this to create a finger joint training system that incorporates human–machine interaction (including a front-end data collector and back-end data analysis software) to further improve the intelligence of rehabilitation medicine and enhance the user experience. In summary, the supramolecular bionic electronic skin proposed in this paper will be more suitable for future needs of human–machine interaction in multi-complex scenarios. PGC electronic skin patch has broad application prospects in the fields of new generation wearable electronic skin, rehabilitation medicine, human–machine interaction, VR/AR and metaverse.

### Supplementary Information

Below is the link to the electronic supplementary material.

Supplementary file1 (PDF 1739 kb)

Supplementary file2 (MP4 14668 kb)

Supplementary file3 (MP4 11942 kb)

Supplementary file4 (MP4 11767 kb)

Supplementary file5 (MP4 4112 kb)
