# Supplementary material for: Skin-Inspired Ultra-Tough Supramolecular Multifunctional Hydrogel Electronic Skin for Human–Machine Interaction
Source: Nanomicro Lett. 2023 Apr 13;15:102. doi: 10.1007/s40820-023-01084-8 (PMC10102281; doi:10.1007/s40820-023-01084-8)
Supplement: Supplementary file 1 — Supplementary file1 (PDF 1739 kb) [file 40820_2023_1084_MOESM1_ESM.pdf]

Supporting Information for

Skin-Inspired Ultra-Tough Supramolecular Multifunctional Hydrogel Electronic Skin for Human-Machine Interaction

Kun Chen[1], Kewei Liang[1], He Liu [1], Ruonan Liu[1], Yiying Liu[1], Sijia Zeng [1], and Ye Tian[1, 2, *]

[1]College of Medicine and Biological Information Engineering, Northeastern University, Shenyang 110169, P. R. China

[2]Foshan Graduate School of Innovation, Northeastern University, Foshan, 528300, P. R. China

*Corresponding author. E-mail: tianye@bmie.neu.edu.cn (Ye Tian)

Supplementary Figures and Tables

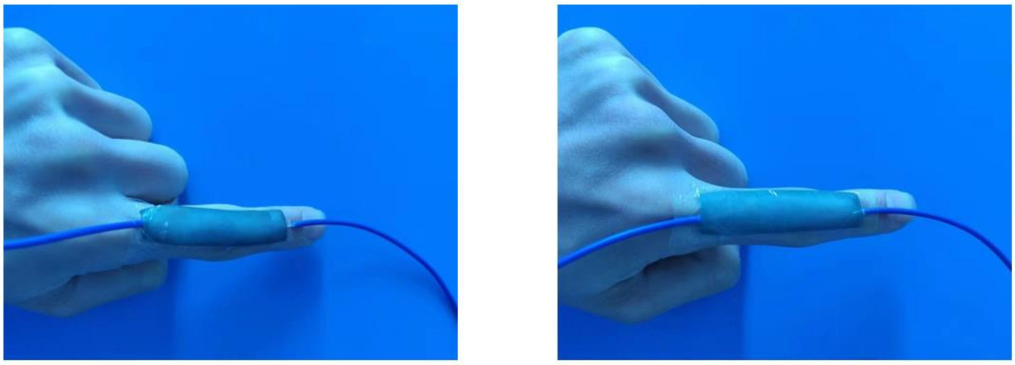

Fig. S1 The protype of PGC sensor

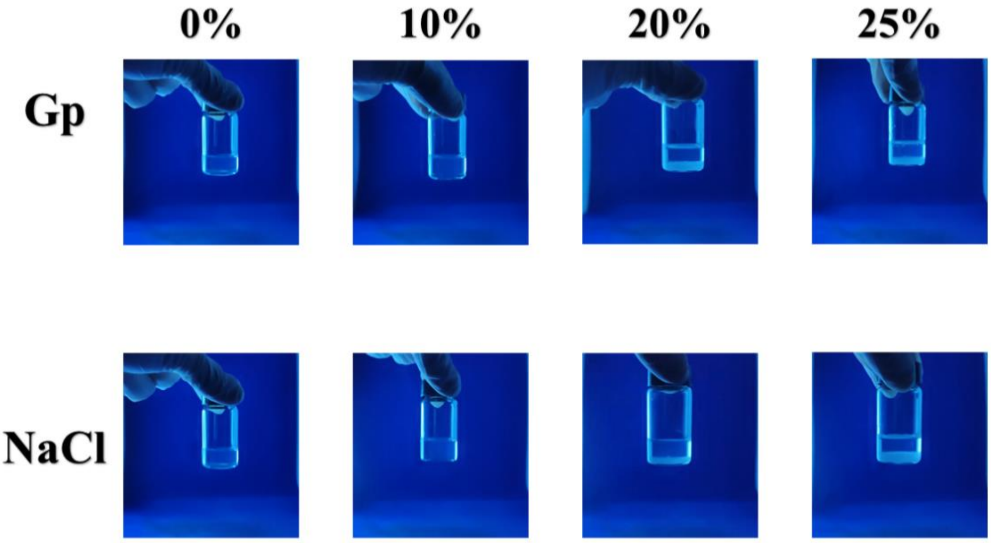

Fig. S2 Phenomena after adding different concentrations of Gp and NaCl (As the concentration of added salt increased, more precipitates can be produced in the PVA solution after the addition of Gp than after the addition of NaCl)

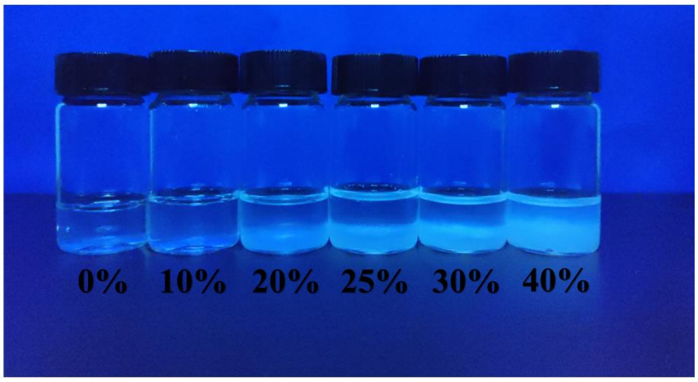

Fig. S3 The reaction of PVA solution after adding different concentrations of Gp

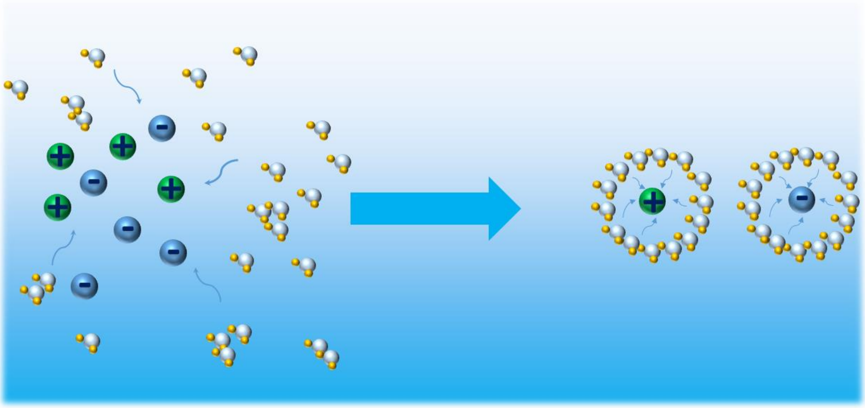

Fig. S4 Microscopic diagram of hydration process

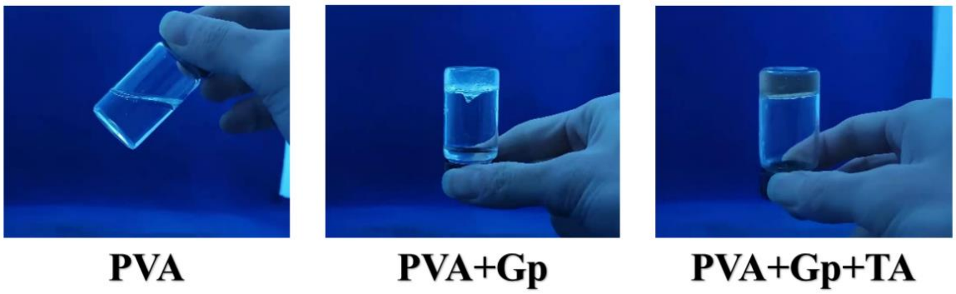

Fig. S5 The morphology of PGP, PGT hydrogel at room temperature after water bath

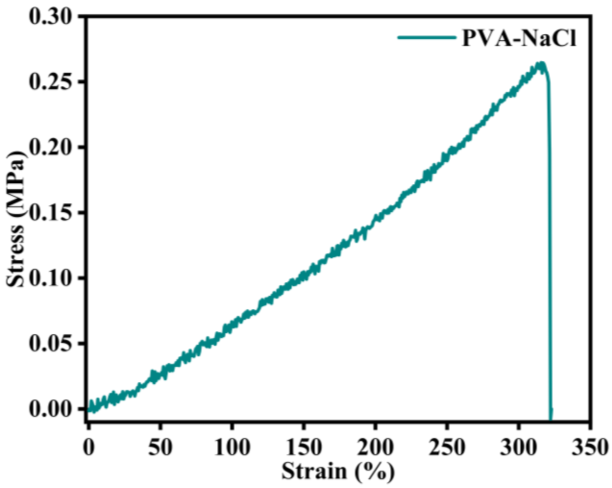

Fig. S6 The stress-strain curves of PVA-NaCl hydrogel

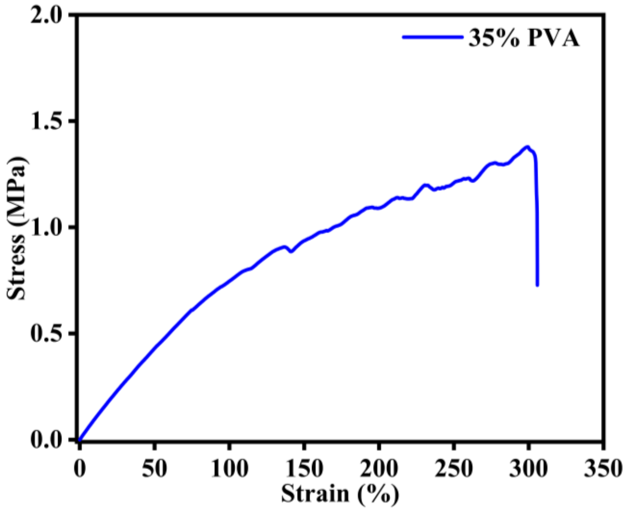

Fig. S7 The stress-strain curves of 35% PVA hydrogel

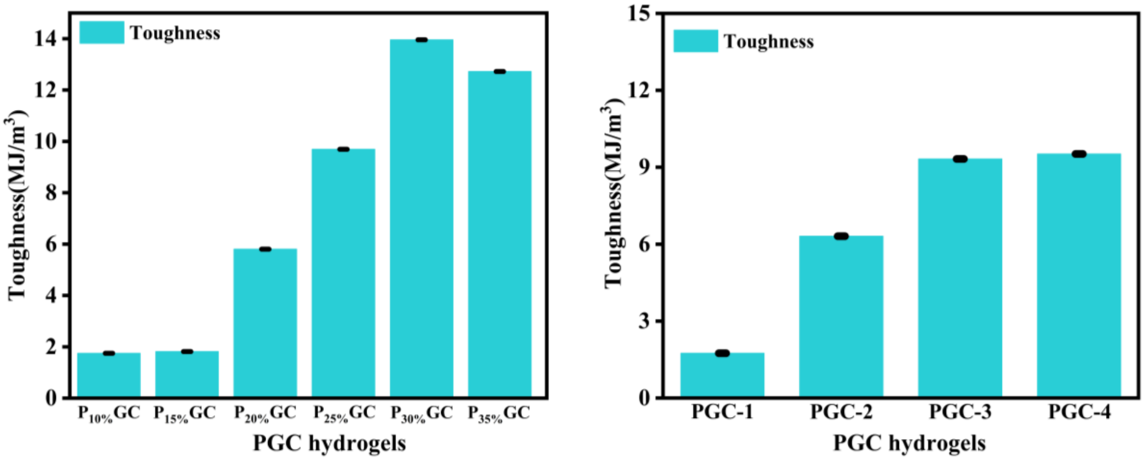

Fig. S8 The toughness of hydrogels (measured from the original hydrogels)

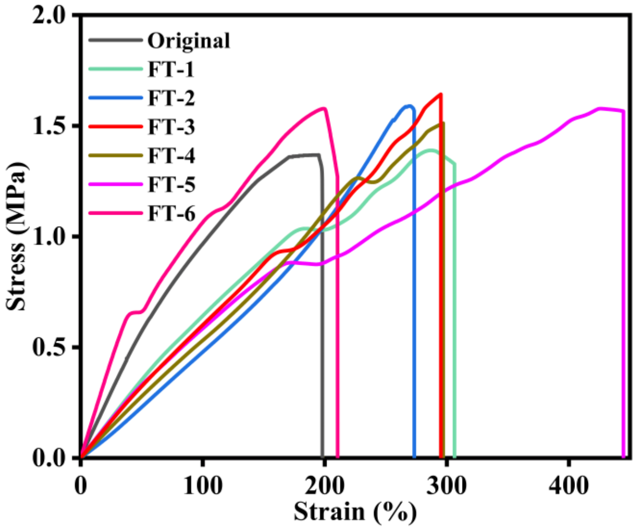

Fig. S9 The stress-strain curves of the original hydrogel and after six recycling cycles (derived from PGC hydrogels with different number of cycles)

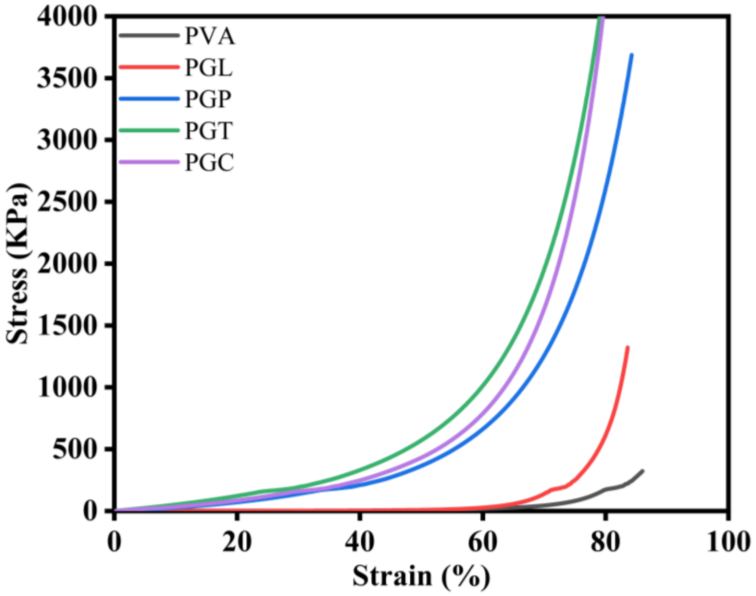

Fig. S10 Compressive strain curve of hydrogels

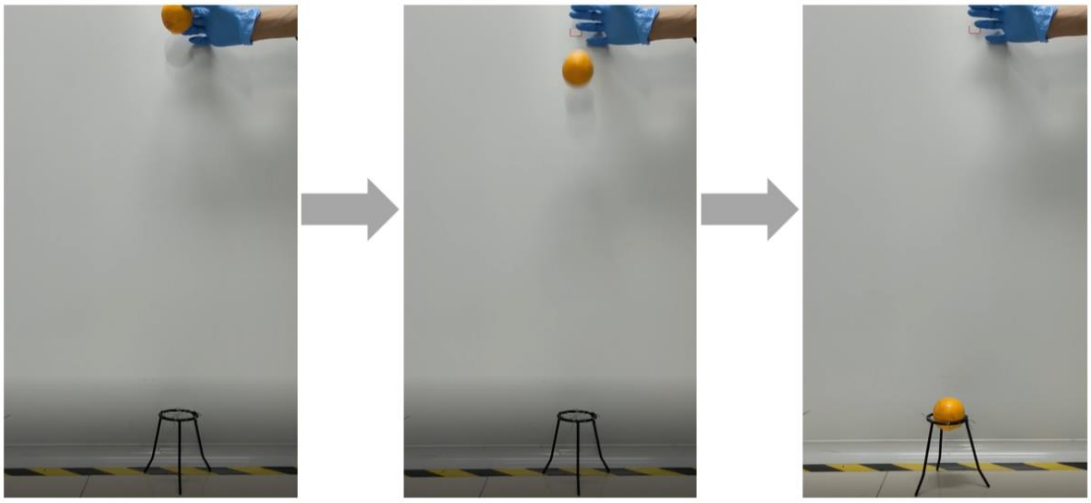

Fig. S11 An orange of 172 g dropped from a height of 1m onto a hydrogel network

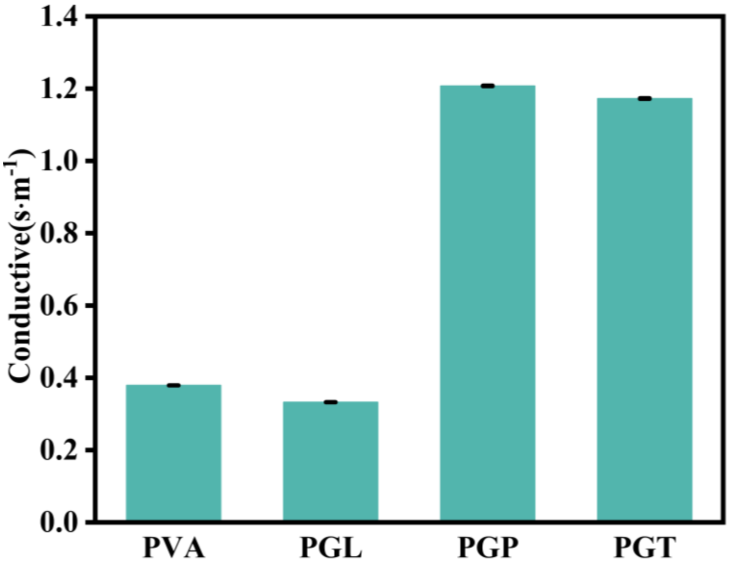

Fig. S12 Comparison of electrical conductivity between different hydrogels

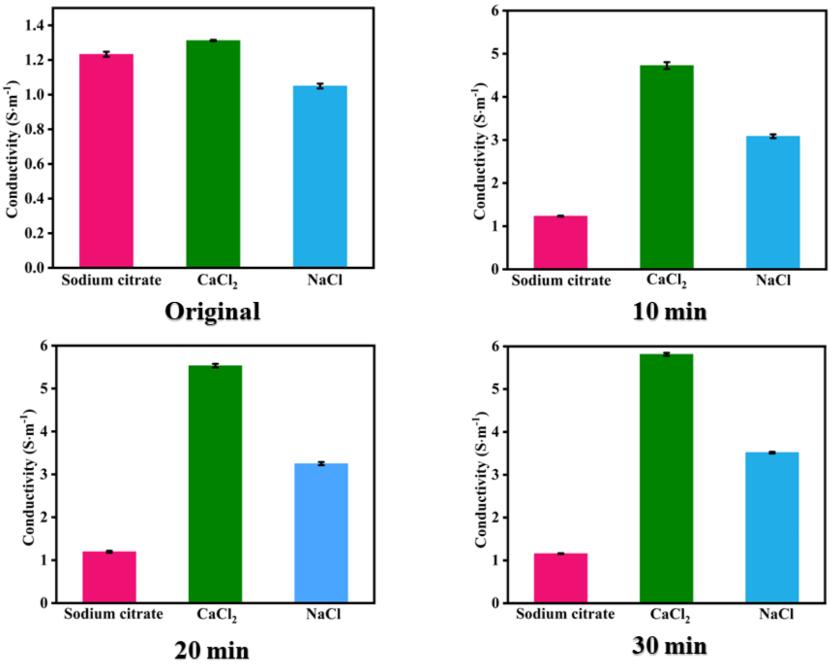

Fig. S13 Conductivity at different times in ionic solutions

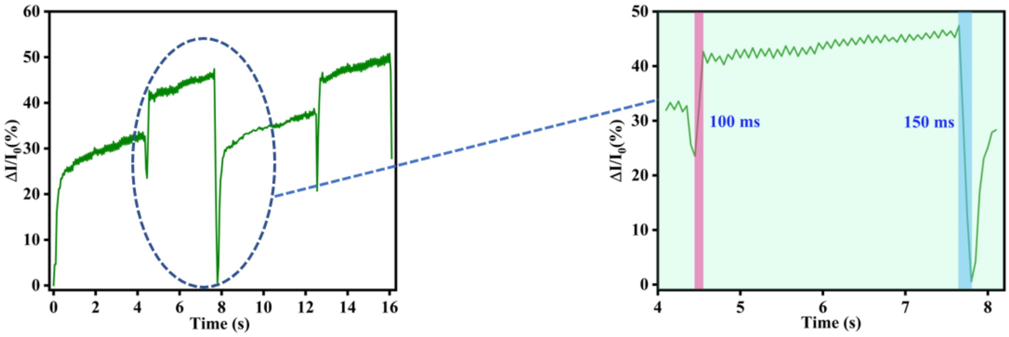

Fig. S14 Response/recovery time of PGC hydrogels during stretch-relaxation

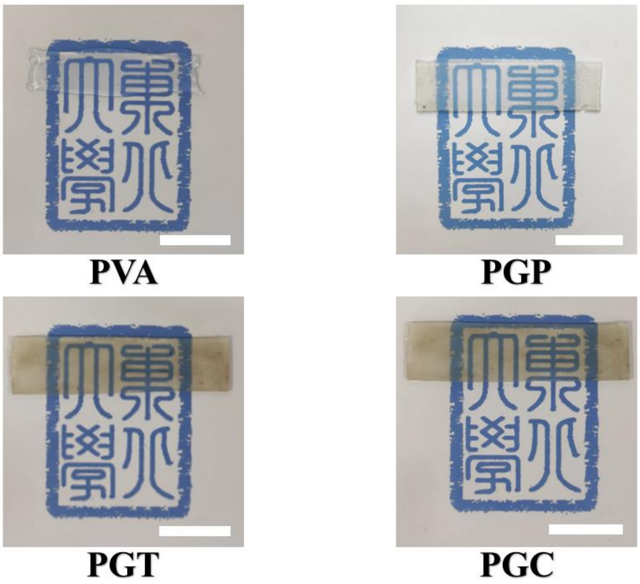

Fig. S15 Transparency of different hydrogel samples. Scale bars: 2 cm

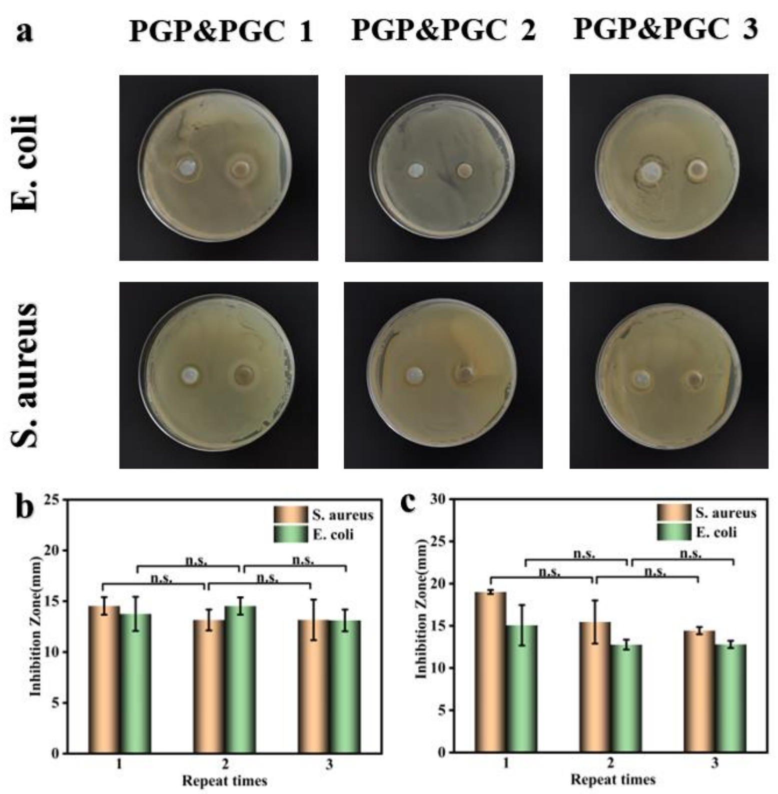

Fig. S16 Antibacterial repeatability of PGP and PGC hydrogels. **a** Inhibition zone photographs and diameters of inhibition circles of **b** PGP and **c** PGC hydrogels (data are depicted as mean ± SD, n.s.: no significant difference, n = 3)

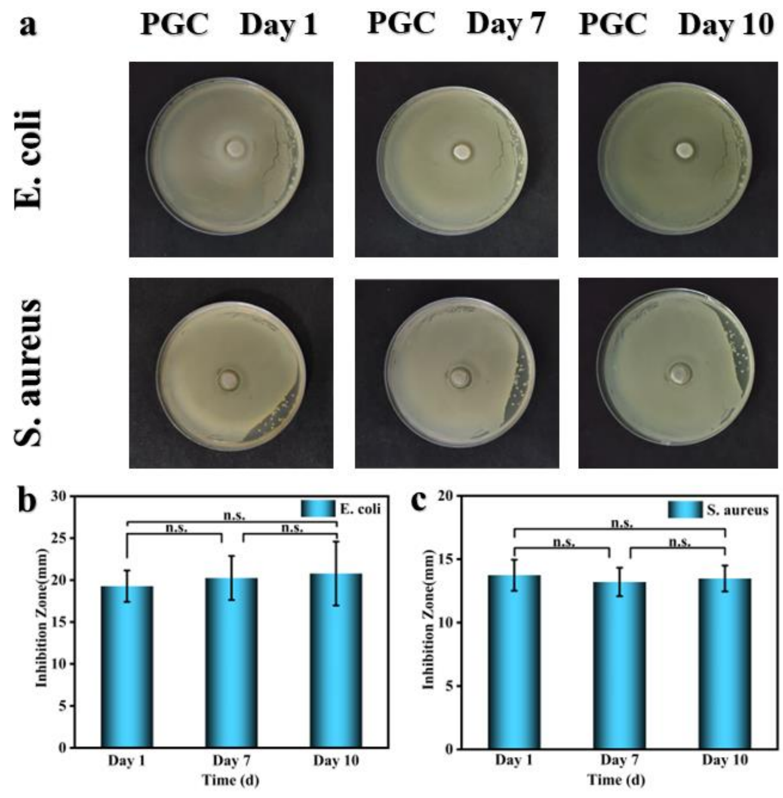

Fig. S17 Antibacterial durability of PGC hydrogel. **a** Inhibition zone photographs and diameters of inhibition circles of **b** E. coli and **c** S. aureus at 1, 7, and 10 days (data are depicted as mean ± SD, n.s.: no significant difference, n = 3)

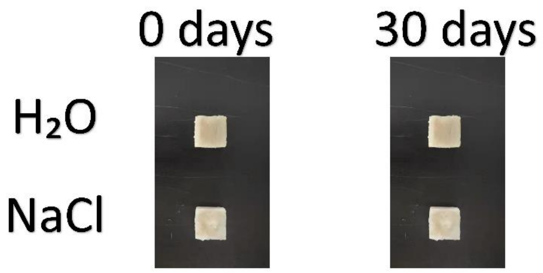

Fig. S18 Before and after volume comparison of hydrogels immersed in deionized water/simulated seawater for 30 days

011001-------Safe arrival
010001-------In progress
010011-------Dangerous
001101-------Special cases
010101-------Physical overdraft
001010-------Low visibility
010010-------Low oxygen level
011111-------Emergency support

Fig. S19 Morse code appendix

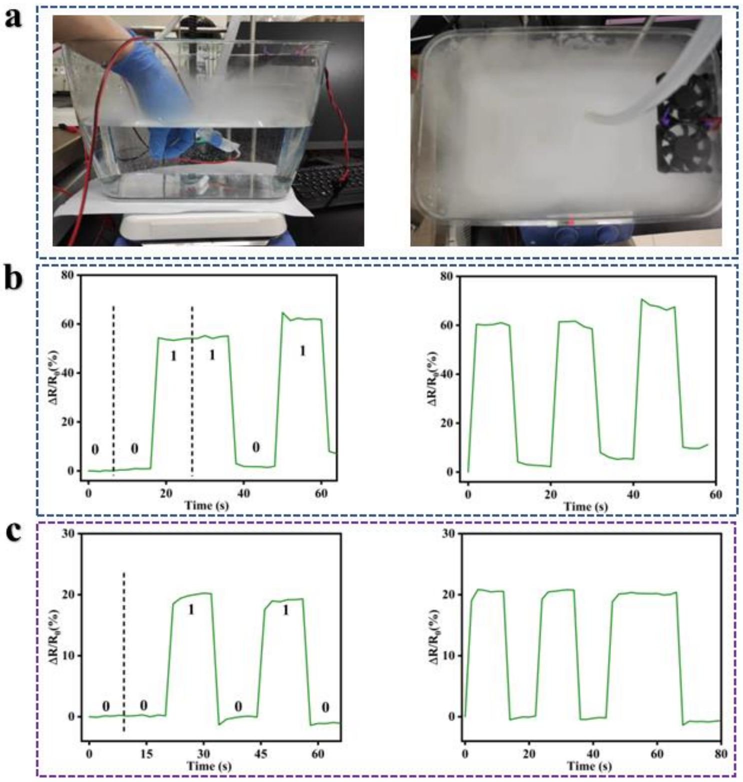

Fig. S20 a Schematic diagram of simulating environmental factors such as humidity and waves. **b** Sensing characterization in water. **c** Sensing characterization in simulated seawater water

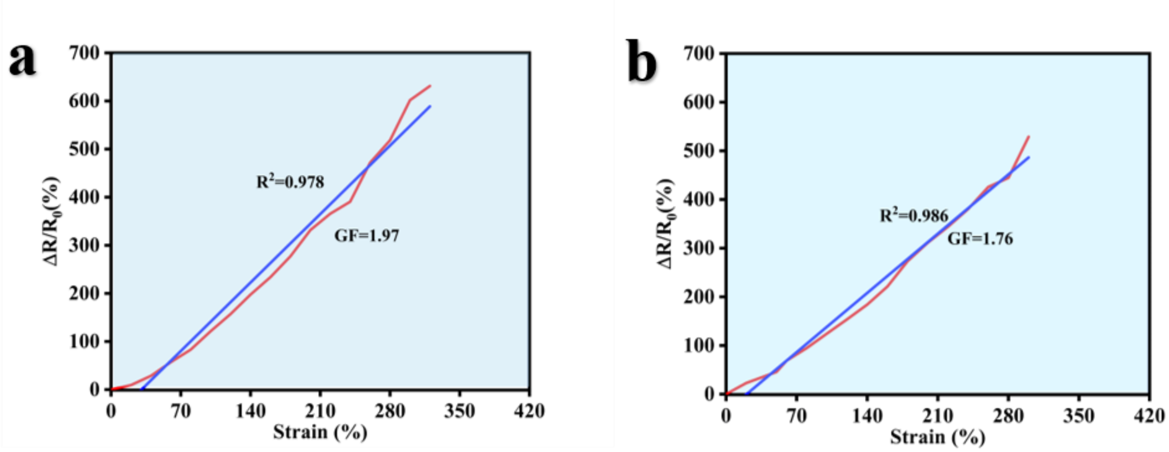

Fig. S21 The GF in **a** water flow and **b** wet environment

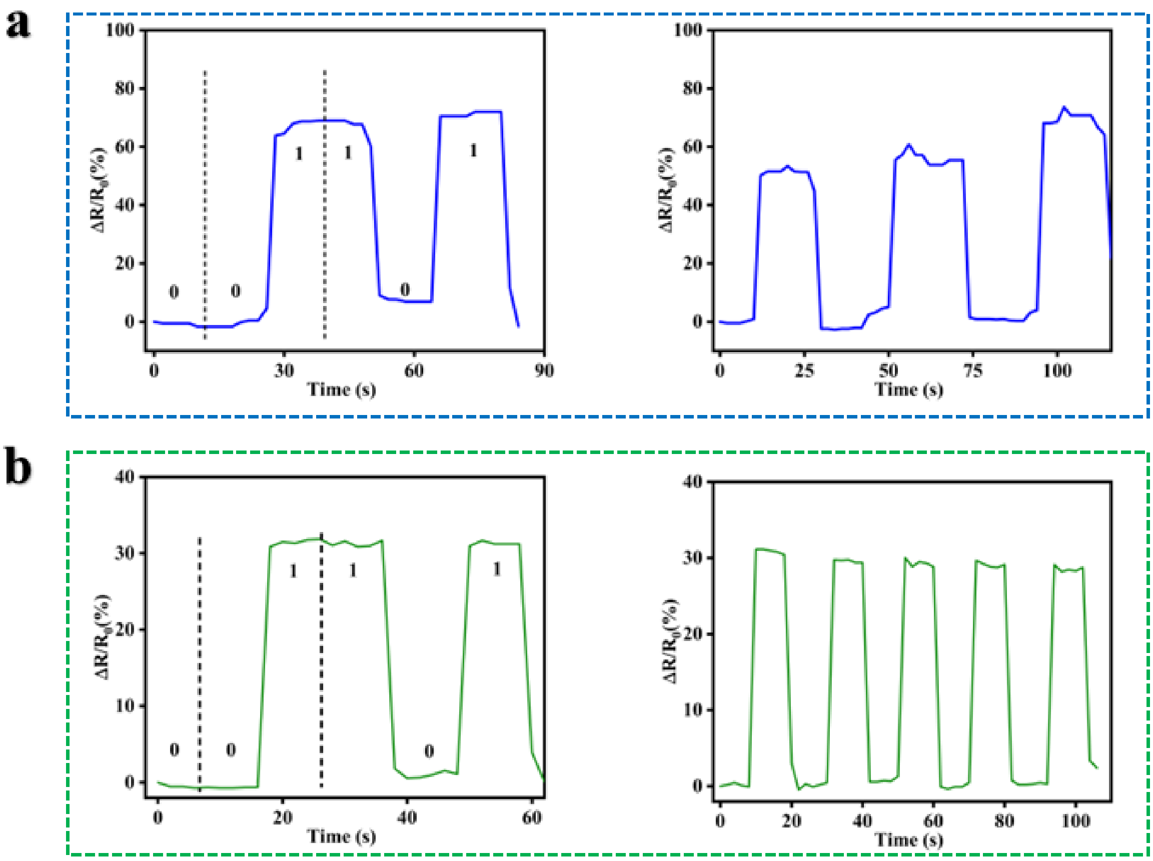

Fig. S22 The stability tests in **a** water and **b** simulated seawater, respectively

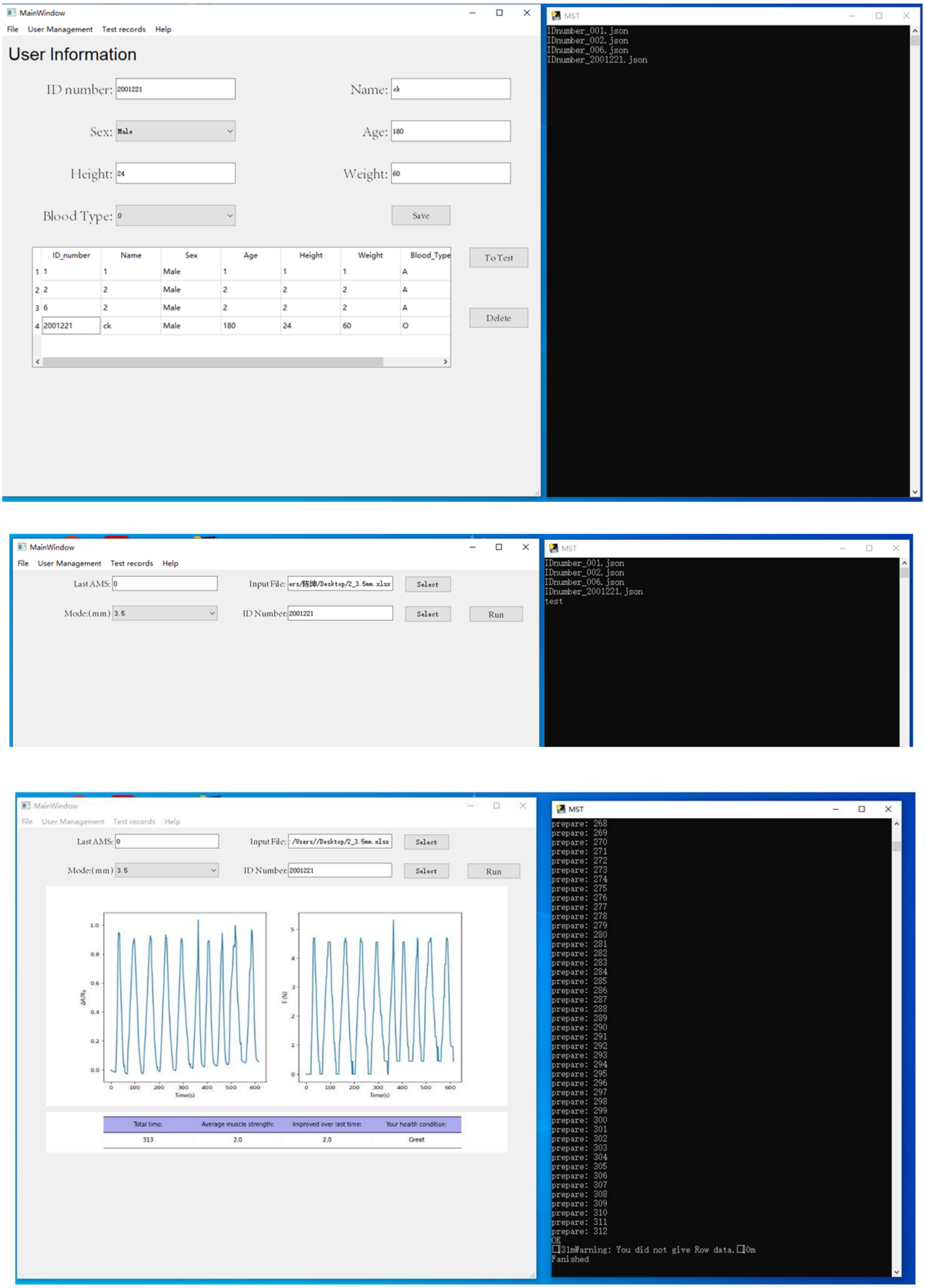

Fig. S23 Human-machine interaction interface design for MST systems

Table S1 Comparison of mechanical strength of our work and recently reported PVA-based hydrogel/hydrogel e-skin

**Materials**	**Mechanical stress**	**Category**	**References**
CNCs-Fe^{3+}/PVA/PVP	2.1MPa	PVA-based	[S1]
CNF/clay/PVA	0.09MPa	PVA-based	[S2]
PAM/PAM/PEI/LiCl	0.8MPa	PVA-based	[S3]
PMZn-GL	0.875MPa	PVA-based	[S4]
Zn-alginate/PVA	0.15MPa	PVA-based	[S5]
PVA-G-PDA-AgNPs	1.174MPa	PVA-based	[S6]
PVA/DMSO/rGO	3.1MPa	PVA-based	[S7]
PVA-CNF	2.1MPa	PVA-based	[S8]
PVA	5.2MPa	PVA-based	[S9]
PVA/SA/NaCl	1.32MPa	PVA-based	[S10]
PVA/(PVA-MA)-g-PNIPAM	2.2MPa	PVA-based	[S11]
PVA/HPC/DMSO/NaCl	4MPa	PVA-based	[S12]
PVA/GC	1.4MPa	PVA-based	[S13]
PVA/PANI/SWCNT	0.47MPa	PVA-based	[S14]
PVA/SiO_2/PEG	0.863MPa	PVA-based	[S15]
PAM/PVA	0.18MPa	PVA-based	[S16]
MXene/PVA/CMC/TA	1.8MPa	PVA-based	[S17]
PVA/PAM/NaCl	0.477MPa	PVA-based	[S18]
PVA-PAANa-PAH	0.86MPa	PVA-based	[S19]
PDMS/CNT	0.09MPa	E-skin	[S20]
PVA/Bn/PEI/MXene	0.0032MPa	E-skin	[S21]
PAAS-MXene	0.07MPa	E-skin	[S22]
GelMA/PEGDA/CNT/PDA	0.02MPa	E-skin	[S23]
PAA-GO-Ca^{2+}	0.74MPa	E-skin	[S24]
NaCl/SA/PAA-PAM	0.27MPa	E-skin	[S25]
PDA@AgNPs/CPHs	0.104MPa	E-skin	[S26]
PVA/Gp/TA/$CaCl^{2+}$	5.79MPa	PVA-based E-skin	This work

Table S2 Comparison of gauge factors (GF) of our work and recently reported gel-based materials

Materials	Conductive materials	Gauge factor	References
k-Carrageenan/PAAM	Na^+	0.63	[S35]
CNCs-Fe^{3+}/PVA/PVP	Fe^{3+}	0.478	[S1]
CNF/clay/PVA	Al^{3+}	1.17	[S2]
GelMA/PEGDA/CNT/PDA	CNT	1.23	[S23]
PVA-G-PDA-AgNPS	Graphene	0.94	[S6]
PAM/AgNPS/PDA/NFC	Ag	0.34	[S31]
PAM/SBMA/PEG/HEA	Zn^{2+}	0.14	[S32]
PVA/CNF	Na^+	1.5	[S8]
PVA/GC	Na^+	1.56	[S13]
SMA/CNFs/PAM	K^+	0.3	[S28]
PVA-PAANa-PAH	Na^+	1.64	[S19]
PVA/Gp/TA/$CaCl_2$	Ca^{2+}	1.73	This work

Table S3 Comparison of response/recovery time of our work and recently reported gel-based materials

Materials	Response time	Recovery time	References
CNF/clay/PVA	240 ms	/	[S2]
PMZn-GL	250 ms	240 ms	[S4]
PVA/DMSO/rGO/GO	250 ms	250 ms	[S7]
PVA/GC	266 ms	250 ms	[S13]
PAM/PVA	150 ms	/	[S16]
PVA/PAM/NaCl	300 ms	/	[S18]
PVA/Bn/PEI/MXene	120 ms	/	[S21]
GelMA/PEGDA/CNT/PDA	180 ms	/	[S23]
NaCl/SA/PAA-PAM	100 ms	100 ms	[S25]
SMA/CNFs/PAM	134 ms	/	[S28]
PAM/SBMA/PEG/HEA	248 ms	280 ms	[S32]
PVA/Gp/TA/$CaCl_2$	100 ms	150 ms	This work

Table S4 Performance control with other research work

	Materials	Stress MPa	Recycle twice	Conductivity S m^{-1}	GF (Linear)	AI interaction	Process	References
S1.	GelMA/PEGDA/CNT/PDA	0-0.02	/	2.63	NO	/	Infrared-light-induced crosslinking	[S23]
S2.	Ag/PAM-Na_3cit	0-2	/	0.98	Yes	/	Free-radical polymerization	[S33]
S3.	PVA/G/PDA/AgNPs	0-1.174	~88%	3.56	NO	/	Repeat freezing cycle	[S6]
S4.	PAM/AgNPs/PDA/NFC	0-0.717	/	/	NO	/	Free-radical polymerization	[S31]
S5.	PVA/DMSO/rGO	0-3.1	/	/	Yes	Pressure Sensing	Repeat freezing cycle	[S7]
S6.	PAA–GO–Ca^{2+}	0-0.7419	/	/	/	Wireless transmission	Free-radical polymerization	[S24]
S7.	NaCl/SA/PAA-PAM	0-0.27	/	2.2	Yes	/	Free-radical polymerization	[S25]
S8.	PAM/SBMA/PEG/HEA	0-1.02	/	/	NO	Wireless transmission	Free-radical polymerization	[S32]
S9.	PVA-CNF	0-2.1	/	3.2	NO	/	Oil bath	[S8]
S10.	PVA	0-5.2	/	/	/	/	Repeat freezing cycle	[S9]
S11.	AG/PAAm/LiCl	0-0.38	/	/	/	/	Ultrasonication	[S30]
S12.	PVA/SA/NaCl	0-1.32	/	3.62	/	/	Repeat freezing cycle	[S10]
S13.	PVA/(PVA-MA)-g-PNIPAM	0-2.2	/	/	/	/	Acid-treatment;Salt induction	[S11]

S14.	PVA/HPC/DMSO/NaCl	0-4	/	3.4	/	/	Repeat freezing cycle	[S12]
S15.	PVA/GC	0-1.4	/	/	Yes	/	Freezing and thawing	[S13]
S16.	PDA@Ag NPs/CPHs	0-0.104	/	/	/	/	Ultra-low temperature freezing	[S26]
S17.	PVA/PANI/SWCNT	0-0.47	/	13.64	/	/	Acid treatment, ultrasonic dispersion	[S14]
S18.	PVA/SiO_2/PEG	0-0.863	/	5.73	/	/	Ultrasound, organic dispersion	[S15]
S19.	PAM/PVA	0-0.18	/	/	/	/	Free-radical polymerization	[S16]
S20.	MXene/PVA/CMC/TA	0-1.8	/	1.5	Yes	Pressure Sensing	Repeat freezing cycle	[S17]
S21.	SA/PAM/NaCl	0-0.75	74%	0.05	NO	/	Free-radical polymerization	[S27]
S22.	SMA/CNFs/PAM	0-0.55	/	4.35	Yes	/	Free-radical polymerization	[S28]
S23.	PVA/PAM/NaCl	0-0.477	/	6.23	NO	/	Free-radical polymerization	[S18]
S24.	PVA-PAANa-PAH	0-0.86	/	0.45	Yes	/	Freeze-thaw;salt precipitation	[S19]
S25.	P(Urea-Ila-$SPMA_b$)-xd	0-2.14	/	3	/	/	Free-radical polymerization	[S29]
S26.	PVA/Gp/TA/$CaCl_2$	0-3	100%	4.72	Yes	Information encryption	One freezing cycle	This work

Table S5 Performance control with other research work

	Materials	Antibacterial	UV protection	Toughness	Transparency	References
S1.	GelMA/PEGDA/CNT/PDA	NO	NO	/	/	[S23]
S2.	Ag/PAM-Na_3cit	Yes	NO	11.95	/	[S33]
S3.	PVA−G−PDA−AgNPs	Yes	NO	/	/	[S6]
S4.	PAM/AgNPs/PDA/NFC	Yes	NO	/	/	[S31]
S5.	PVA/DMSO/rGO/GO	NO	NO	/	/	[S7]
S6.	PAA–GO–Ca^{2+}	NO	NO	9.73	/	[S24]
S7.	NaCl/SA/PAA-PAM	NO	NO	/	/	[S25]
S8.	PAM/SBMA/PEG/HEA	NO	NO	/	/	[S32]
S9.	PVA-CNF	NO	NO	5.25	90%	[S8]
S10.	PVA	NO	NO	/	/	[S9]
S11.	AG/PAAm/LiCl	NO	NO	4.4	/	[S30]
S12.	PVA/SA/NaCl	NO	NO	/	/	[S10]
S13.	PVA/(PVA-MA)-g-PNIPAM	NO	NO	10	/	[S11]

S14.	PVA/HPC/DMSO/NaCl	NO	NO	/	/	[S12]
S15.	PVA/GC	NO	NO	3.2	90%	[S13]
S16.	PDA@Ag NPs/CPHs	Yes	NO	/	/	[S26]
S17.	PVA/PANI/SWCNT	NO	NO	/	/	[S14]
S18.	PVA/SiO_2/PEG	NO	NO	/	/	[S15]
S19.	PAM/PVA	NO	NO	/	>90%	[S16]
S20.	MXene/PVA/CMC/TA	NO	NO	6.24	/	[S17]
S21.	SA/PAM/NaCl	NO	NO	4.77	99.6%	[S27]
S22.	SMA/CNFs/PAM	Yes	NO	0.2	/	[S28]
S23.	PVA/PAM/NaCl	NO	NO	2.5	/	[S18]
S24.	PVA-PAANa-PAH	NO	NO	2.25	79.04%	[S19]
S25.	P(Urea-Ila-$SPMA_b$)-xd	NO	NO	6.7	/	[S29]
S26.	PVA/Gp/TA/$CaCl_2$	Yes	Yes	8.178	>60%	This work

Partial code demonstration of the decryption procedure:

```
import argparse
import matplotlib
import matplotlib.pyplot as plt
import numpy as np
import pandas as pd
from matplotlib.backends.backend_agg import FigureCanvasAgg
import matplotlib.patheffects as path_effects
import matplotlib
from yaml import parse
from typing import Dict
import pytab
import PIL.Image as Image
from matplotlib import font_manager
my_font=font_manager. FontProperties(fname="C:\Windows\Fonts\msyh.ttc")
import cv2
fps =5#
fourcc = cv2.VideoWriter_fourcc(*'XVID')
dic= {"011001":"Safe arrival","010001":"In progress","010011":"Dangerous","001101":"Special cases","010101":"Physical overdraft",
"001010":"Low visibility","010010":"Low oxygen level","011111":"Emergency support"}
parser=argparse.ArgumentParser()
parser.add_argument("--save_path", type=str,default="D:/project/leetcode/chenkun/save/out.jpg")
parser.add_argument("--save_path_video", type=str,default="D:/project/leetcode/chenkun/save/out.mp4")
parser.add_argument("--input_path", type=str,default='D:/project/leetcode/chenkun/code/water/011001.xlsx')
opt=parser.parse_args()
video_path=opt.save_path_video
videoWriter = cv2.VideoWriter(video_path, fourcc, fps, (720,540))
data = pd.read_excel(opt.input_path, sheet_name=0, header=0)
Y=np.array(data["Y"])
max_y=np.max(Y)
min_y=np.min(Y)
```

```
cent=(max_y+min_y)/2
jieguo= []
temp_0=0
temp_1=0
for i in range(len(Y)):
   if Y[i]>cent:
      if temp_0! = 0:
         a=temp_0/5
         for j in range(round(temp_0/5)):
            jieguo.append(0)
         temp_0=0
      temp_1=temp_1+1
   else:
      if temp_1! = 0:
         for j in range(round(temp_1/5)):
            jieguo.append(1)
         temp_1=0
      temp_0=temp_0+1
if temp_0! = 0:
   for j in range(round(temp_0/5)):
            jieguo.append(0)
if temp_1! = 0:
   for j in range(round(temp_1/5)):
            jieguo.append(1)
```

Movie S1 The phenomenon of aggregation and sinking of PVA aqueous solution

Movie S2 Underwater sensing and encryption and decryption of information

Movie S3 The use of finger joint rehabilitation trainers

Movie S4 Demonstration of the operation of the MST software

Supplementary References

[S1] Y.-J. Liu, W.-T. Cao, M.-G. Ma, P. Wan, Ultrasensitive wearable soft strain sensors of conductive, self-healing, and elastic hydrogels with synergistic “soft and hard” hybrid networks. ACS Appl. Mater. Interfaces **9**(30), 25559-25570 (2017). https://doi.org/10.1021/acsami.7b07639

[S2] K. Hu, P. He, Z. Zhao, L. Huang, K. Liu et al., Nature-inspired self-powered cellulose nanofibrils hydrogels with high sensitivity and mechanical adaptability.

Carbohydr. Polym. **264**, 117995 (2021). https://doi.org/10.1016/j.carbpol.2021.117995

[S3] Y. Wang, L. Sun, G. Chen, H. Chen, Y. Zhao, Structural color ionic hydrogel patches for wound management. ACS Nano **17**(2), 1437-1447 (2022). https://doi.org/10.1021/acsnano.2c10142

[S4] Y. Feng, H. Liu, W. Zhu, L. Guan, X. Yang et al., Muscle-inspired MXene conductive hydrogels with anisotropy and low-temperature tolerance for wearable flexible sensors and arrays. Adv. Funct. Mater. **31**(46), 2105264 (2021). https://doi.org/10.1002/adfm.202105264

[S5] F. Mo, Y. Huang, Q. Li, Z. Wang, R. Jiang et al., A highly stable and durable capacitive strain sensor based on dynamically super-tough hydro/organo-gels. Adv. Funct. Mater. **31**(28), 2010830 (2021). https://doi.org/10.1002/adfm.202010830

[S6] L. Fan, J. Xie, Y. Zheng, D. Wei, D. Yao et al., Antibacterial, self-adhesive, recyclable, and tough conductive composite hydrogels for ultrasensitive strain sensing. ACS Appl. Mater. Interfaces **12**(19), 22225-22236 (2020). https://doi.org/10.1021/acsami.0c06091

[S7] H. Chen, J. Huang, J. Liu, J. Gu, J. Zhu et al., High toughness multifunctional organic hydrogels for flexible strain and temperature sensor. J. Mater. Chem. A **9**(40), 23243-23255 (2021). https://doi.org/10.1039/D1TA07127K

[S8] Y. Ye, Y. Zhang, Y. Chen, X. Han, F. Jiang, Cellulose nanofibrils enhanced, strong, stretchable, freezing-tolerant ionic conductive organohydrogel for multi-functional sensors. Adv. Funct. Mater. **30**(35), 2003430 (2020). https://doi.org/10.1002/adfm.202003430

[S9] S. Lin, J. Liu, X. Liu, X. Zhao, Muscle-like fatigue-resistant hydrogels by mechanical training. Proc. Natl. Acad. Sci. **116**(21), 10244-10249 (2019). https://doi.org/10.1073/pnas.1903019116

[S10] X. Jiang, N. Xiang, H. Zhang, Y. Sun, Z. Lin et al., Preparation and characterization of poly(vinyl alcohol)/sodium alginate hydrogel with high toughness and electric conductivity. Carbohydr. Polym. **186**, 377-383 (2018). https://doi.org/10.1016/j.carbpol.2018.01.061

[S11] M. Hua, D. Wu, S. Wu, Y. Ma, Y. Alsaid et al., 4D printable tough and thermoresponsive hydrogels. ACS Appl. Mater. Interfaces **13**(11), 12689-12697 (2021). https://doi.org/10.1021/acsami.0c17532

[S12] Y. Zhou, C. Wan, Y. Yang, H. Yang, S. Wang et al., Highly stretchable, elastic, and ionic conductive hydrogel for artificial soft electronics. Adv. Funct. Mater. **29**(1), 1806220 (2019). https://doi.org/10.1002/adfm.201806220

[S13] X.-J. Zha, S.-T. Zhang, J.-H. Pu, X. Zhao, K. Ke et al., Nanofibrillar poly(vinyl alcohol) ionic organohydrogels for smart contact lens and human-interactive sensing. ACS Appl. Mater. Interfaces **12**(20), 23514-23522 (2020). https://doi.org/10.1021/acsami.0c06263

[S14] Y. Guo, K. Zheng, P. Wan, A flexible stretchable hydrogel electrolyte for healable all-in-one configured supercapacitors. Small **14**(14), 1704497 (2018). https://doi.org/10.1002/smll.201704497

[S15] X. Fan, J. Liu, Z. Song, X. Han, Y. Deng et al., Porous nanocomposite gel polymer electrolyte with high ionic conductivity and superior electrolyte retention capability for long-cycle-life flexible zinc-air batteries. Nano Energy **56**, 454-462 (2019). https://doi.org/10.1016/j.nanoen.2018.11.057

[S16] G. Ge, Y. Zhang, J. Shao, W. Wang, W. Si et al., Stretchable, transparent, and self-patterned hydrogel-based pressure sensor for human motions detection. Adv. Funct. Mater. **28**(32), 1802576 (2018). https://doi.org/10.1002/adfm.201802576

[S17] D. Kong, Z.M. El-Bahy, H. Algadi, T. Li, S.M. El-Bahy et al., Highly sensitive strain sensors with wide operation range from strong MXene-composited polyvinyl alcohol/sodium carboxymethylcellulose double network hydrogel. Adv. Compos. Hybrid Mater. **5**(3), 1976-1987 (2022). https://doi.org/10.1007/s42114-022-00531-1

[S18] G. Chen, J. Huang, J. Gu, S. Peng, X. Xiang et al., Highly tough supramolecular double network hydrogel electrolytes for an artificial flexible and low-temperature tolerant sensor. J. Mater. Chem. A **8**(14), 6776-6784 (2020). https://doi.org/10.1039/D0TA00002G

[S19] W.J. Yang, R. Zhang, X. Guo, R. Ma, Z. Liu et al., Supramolecular polyelectrolyte hydrogel based on conjoined double-networks for multifunctional applications. J. Mater. Chem. A **10**(44), 23649-23665 (2022). https://doi.org/10.1039/D2TA05530A

[S20] Y. Cheng, Y. Zhou, R. Wang, K.H. Chan, Y. Liu et al., An elastic and damage-tolerant dry epidermal patch with robust skin adhesion for bioelectronic interfacing. ACS Nano **16**(11), 18608-18620 (2022). https://doi.org/10.1021/acsnano.2c07097

[S21] W. Peng, L. Han, H. Huang, X. Xuan, G. Pan et al., A direction-aware and ultrafast self-healing dual network hydrogel for a flexible electronic skin strain sensor. J. Mater. Chem. A **8**(48), 26109-26118 (2020). https://doi.org/10.1039/D0TA08987G

[S22] J. Luo, C. Sun, B. Chang, Y. Jing, K. Li et al., MXene-enabled self-adaptive hydrogel interface for active electroencephalogram interactions. ACS Nano **16**(11), 19373-19384 (2022). https://doi.org/10.1021/acsnano.2c08961

[S23] H. Tang, Y. Li, B. Chen, X. Chen, Y. Han et al., In situ forming epidermal bioelectronics for daily monitoring and comprehensive exercise. ACS Nano **16**(11), 17931-17947 (2022). https://doi.org/10.1021/acsnano.2c03414

[S24] Y. Wang, Q. Chang, R. Zhan, K. Xu, Y. Wang et al., Tough but self-healing and 3d printable hydrogels for e-skin, e-noses and laser controlled actuators. J. Mater. Chem. A **7**(43), 24814-24829 (2019). https://doi.org/10.1039/C9TA04248B

[S25] H. Huang, L. Han, X. Fu, Y. Wang, Z. Yang et al., Multiple stimuli responsive and identifiable zwitterionic ionic conductive hydrogel for bionic electronic skin. Adv. Electron. Mater. **6**(7), 2000239 (2020). https://doi.org/10.1002/aelm.202000239

[S26] Y. Zhao, Z. Li, S. Song, K. Yang, H. Liu et al., Skin-inspired antibacterial conductive hydrogels for epidermal sensors and diabetic foot wound dressings. Adv. Funct. Mater. **29**(31), 1901474 (2019). https://doi.org/10.1002/adfm.201901474

[S27] X. Zhang, N. Sheng, L. Wang, Y. Tan, C. Liu et al., Supramolecular nanofibrillar hydrogels as highly stretchable, elastic and sensitive ionic sensors. Mater. Horiz. **6**(2), 326-333 (2019). https://doi.org/10.1039/C8MH01188E

[S28] X. Qu, S. Wang, Y. Zhao, H. Huang, Q. Wang et al., Skin-inspired highly stretchable, tough and adhesive hydrogels for tissue-attached sensor. Chem. Eng. J. **425**, 131523 (2021). https://doi.org/10.1016/j.cej.2021.131523

[S29] T. Long, Y. Li, X. Fang, J. Sun, Salt-mediated polyampholyte hydrogels with high mechanical strength, excellent self-healing property, and satisfactory electrical conductivity. Adv. Funct. Mater. **28**(44), 1804416 (2018). https://doi.org/10.1002/adfm.201804416

[S30] L. Fang, Z. Cai, Z. Ding, T. Chen, J. Zhang et al., Skin-inspired surface-microstructured tough hydrogel electrolytes for stretchable supercapacitors. ACS Appl. Mater. Interfaces **11**(24), 21895-21903 (2019). https://doi.org/10.1021/acsami.9b03410

[S31] S. Wang, J. Xiang, Y. Sun, H. Wang, X. Du et al., Skin-inspired nanofibrillated cellulose-reinforced hydrogels with high mechanical strength, long-term antibacterial, and self-recovery ability for wearable strain/pressure sensors. Carbohydr. Polym. **261**, 117894 (2021). https://doi.org/10.1016/j.carbpol.2021.117894

[S32] H. Wang, X. Li, Y. Ji, J. Xu, Z. Ye et al., Highly transparent, mechanical, and self-adhesive zwitterionic conductive hydrogels with polyurethane as a cross-linker for wireless strain sensors. J. Mater. Chem. B **10**(15), 2933-2943 (2022). https://doi.org/10.1039/D2TB00157H

[S33] J. Jian, Y. Xie, S. Gao, Y. Sun, C. Lai et al., A skin-inspired biomimetic strategy to fabricate cellulose enhanced antibacterial hydrogels as strain sensors. Carbohydr. Polym. **294**, 119760 (2022). https://doi.org/10.1016/j.carbpol.2022.119760

[S34] C. Shao, M. Wang, L. Meng, H. Chang, B. Wang et al., Mussel-inspired cellulose nanocomposite tough hydrogels with synergistic self-healing, adhesive, and strain-sensitive properties. Chem. Mater. **30**(9), 3110-3121 (2018). https://doi.org/10.1021/acs.chemmater.8b01172

[S35] S. Liu, L. Li, Ultrastretchable and self-healing double-network hydrogel for 3D printing and strain sensor. ACS Appl. Mater. Interfaces **9**(31), 26429-26437 (2017). https://doi.org/10.1021/acsami.7b07445

[S36] R.A. Li, T. Fan, G. Chen, H. Xie, B. Su et al., Highly transparent, self-healing conductive elastomers enabled by synergistic hydrogen bonding interactions. Chem. Eng. J. **393**, 124685 (2020). https://doi.org/10.1016/j.cej.2020.124685
